# Gut microbiota profile of Indonesian stunted children and children with normal nutritional status

**DOI:** 10.1371/journal.pone.0245399

**Published:** 2021-01-26

**Authors:** Ingrid S. Surono, Dian Widiyanti, Pratiwi D. Kusumo, Koen Venema

**Affiliations:** 1 Food Technology Department, Faculty of Engineering, Bina Nusantara University, Jakarta, Indonesia; 2 Department of Microbiology, Faculty of Medicine, YARSI University, Jakarta, Indonesia; 3 Department of Biomedic, Faculty of Medicine, Universitas Kristen Indonesia, Jakarta, Indonesia; 4 Centre for Healthy Eating & Food Innovation, Maastricht University—Campus Venlo, Venlo, The Netherlands; Washington State University - Spokane, UNITED STATES

## Abstract

The gut microbiota has been shown to play a role in energy metabolism of the host. Dysbiosis of the gut microbiota may predispose to obesity on the one hand, and stunting on the other. The aim of the study was to study the difference in gut microbiota composition of stunted Indonesian children and children of normal nutritional status between 3 and 5 years. Fecal samples and anthropometric measurements, in addition to economic and hygiene status were collected from 78 stunted children and 53 children with normal nutritional status in two regions in Banten and West Java provinces: Pandeglang and Sumedang, respectively. The gut microbiota composition was determined by sequencing amplicons of the V3-V4 region of the 16S rRNA gene. The composition was correlated to nutritional status and anthropometric parameters. Macronutrient intake was on average lower in stunted children, while energy-loss in the form of short-chain fatty acids (SCFA) and branched-chain fatty acids (BCFA) appeared to be higher in stunted children. In stunted children, at the phylum level the relative abundance of Bacteroidetes (44.4%) was significantly lower than in normal children (51.3%; *p*-value 2.55*10^−4^), while Firmicutes was significantly higher (45.7% vs. 39.8%; *p*-value 5.89*10^−4^). At the genus level, overall *Prevotella* 9 was the most abundant genus (average of 27%), and it was significantly lower in stunted children than in normal children (23.5% vs. 30.5%, respectively; *q*-value 0.059). Thirteen other genera were significantly different between stunted and normal children (*q*-value < 0.1), some of which were at low relative abundance and present in only a few children. *Prevotella* 9 positively correlated with height (in line with its higher relative abundance in normal children) and weight. In conclusion, *Prevotella* 9, which was the most abundant genus in the children, was significantly lower in stunted children. The abundance of *Prevotella* has been correlated with dietary fibre intake, which was lower in these stunted children. Since fibres are fermented by the gut microbiota into SCFA, and these SCFA are a source of energy for the host, increasing the proportion of *Prevotella* in stunted children may be of benefit. Whether this would prevent the occurrence of stunting or even has the potential to revert it, remains to be seen in follow up research.

## Introduction

According to the World Health Organization (WHO), stunting is defined as being too short for the child’s age, more precisely when the height-for-age Z-score is more than 2 standard deviations below the WHO Child Growth reference standard median [1]. This is mostly due to poor nutritional intake [2], and has become a major health problem in the world, and it is a cyclic process, where a mother, who experienced stunting in childhood, will likely bear a stunted child [3]. The 2020 data from UNICEF/WHO/World Bank Group [4] reveals that more than one-fifth of children under five-years-old, approximately 144 million worldwide, were stunted due to chronic malnutrition, and 54.3% of those were found in Asia, and 39.9% in Africa. This stunting was largely irreversible after the child’s second birthday.

Stunting has been identified as a major global health priority [5], and the WHO has set the target to reduce stunting by 40% between 2010 and 2025, and improving the identification, measurement and understanding of stunting is part of recommended actions in scaling up the prevention [6]. Although the equation of stunting with malnutrition and chronic infection is commonly acknowledged, there is also some evidence that the link with malnutrition may not always be valid and thus that stunting is not always a synonym of malnutrition, as evidenced in a population of Indonesian children [7].

Indonesia is considered to have a high prevalence of stunting. According to the global nutrition report [8], Indonesia experienced a slight decrease in stunting from 42.4% in 2001 to 36.4% in 2013. Based on current data, the case of stunting has been decreasing further to 30.8% in 2018 [9] and to 27.7% in 2019 [8], but still amounts to 4.6 million children in 2019. Although there is a trend for a gradual decrease, the burden of stunting in Indonesia is still above the rate of stunted children in the Southeast Asia region (24.7%) [8].

Childhood malnutrition is an important health problem in developing countries, and the children may suffer from delayed growth and neurodevelopmental impairment, with consequences of deficiencies in energy, proteins, vitamins (e.g. vitamin A), minerals (e.g. zinc), essential fatty acids and other vital nutritional components (e.g. iodine) [2]. The linear growth retardation in these children already begins *in utero* and continues into infancy and early childhood [10].

The gut microbiota has close links to food digestion, absorption and intestinal function. Persistent undernutrition in childhood will alter the normal (healthy) composition of the intestinal (or gut) microbiota, leading to dysbiosis [11]. *Vice versa*, gut microbiota dysbiosis is associated with malnutrition and reduced plasma essential amino acid levels [12]. Also, there is growing evidence that the gut microbiota influences weight regulation, both in obesity [13] and in anorexia nervosa [14]. Metabolites produced by the gut microbiota have been shown to play a role in weight regulation, particularly the short-chain fatty acids (SCFA) [15, 16]. Also, in stunting, an altered gut microbiota is linked to the pathophysiology of stunting. This alteration may even be detected prior to actually observing stunted growth between 6 and 23 months of life [17]. In the context of stunting, is has been observed that Enterobacteriaceae (of the Proteobacteria phylum, which is often associated with human pathogenicity) are increased in concordance with impaired digestion/absorption and localized gut inflammation [18, 19]. Standard nutritional supplementation in a cohort of 343 Bangladesh children suffering from severe acute malnutrition accomplished improved growth and showed that gut microbiota profiles correlated with biomarkers of growth [20].

The gut microbiota differs, amongst many other variables, with age and with diet. It is individual and regional specific, with different compositions in Asia, Europe, US and Africa [21–24], partly correlated to differences in diets for Asian children [21]. The profile of fecal microbiota of apparently healthy and the microbiota of stunted Indonesian children needs to be explored to find out how gut microbial community structure changes with nutritional status. In 2019 in Indonesia, the Pandeglang district in Banten province and Sumedang district in West Java province were amongst the highest in stunting prevalence, with about 34.0%, and 24.4%, respectively [8]. Ironically, both districts are close to capital cities in Indonesia. Hence, those two districts were selected in this study.

The aim of the study was to explore the interrelationships between the gut microbiota profile and the nutritional status of children, for identification of the key differential microbial groups. This information can be used to correct the observed dysbiosis in these stunted children by optimal interventions to manage severe acute malnutrition, through proper dietary interventions, which in turn modulate the gut microbiota.

## Materials & methods

### Study design and population

A cross-sectional study was conducted on children aged 3–5 years with stunting (*n* = 78) and normal nutritional status (*n* = 53), at two locations, Pandeglang, Banten province, and Sumedang, West Java province, Indonesia. The protocol was approved by the Research Ethics Committee of the Research Institute of YARSI University (dossier No. 004/KEP-UY/BIA/I/2020). Written informed consent was obtained from parents or guardians of the children in the presence of a third person. The nutritional status of each child included in this study was quantified using the WHO recommended three nutritional Z-scores namely, height for age (referred to in this study as Z-score 1); weight for age (referred to as Z-score 2) and weight for height (referred to as Z-score 3). The dietary intake was recorded from 24 hour food-recall and calculated for energy and macronutrient intake using the Nutrisurvey 2007 application (www.nutrisurvey.de). Fecal samples were collected on site from stunted children and children with normal nutritional status and kept in a cooler with ice-packs, and shipped to the lab in Jakarta on dry ice. In the lab, 0.5 g of the feces was mixed with 4.5 ml of Zymo buffer (Baseclear, Leiden, the Netherlands) and kept at room-temperature prior to extraction of DNA.

A structured questionnaire was used for face-to-face interviews with the respective child’s mother or guardian to collect sociodemographic information and feeding practice. In addition, age and anthropometric measurements (height, weight) based on Department of Health Ministry of Indonesia Regulation were recorded. For stunting, the thresholds for height-for-age are: ‘severely stunted’ (<-3 SD); ‘stunted’ (-3 SD to < -2 SD); ‘normal’ (-2 SD to +3 SD); ‘tall’ (> +3 SD). Furthermore, in order to obtain an overall measure of the nutritional status of these children, the children were classified in weight-for-height categories: ‘severely wasted’ (<-3 SD); ‘wasted’ (-3 SD to < -2 SD); ‘normal’ (-2 SD to +1 SD); ‘possible risk of overweight’ (+1 SD to +2 SD); ‘over weight’ (> +2 SD to +3 SD); ‘obese’ (> +3 SD) [1].

### SCFA and BCFA measurements by GC-MS

Feces aliquots of 1 g of were transferred into 25 ml plastic vials, to which 3 ml ethyl acetate and 3 ml formic acid were added. The samples were homogenized on a vortex and then centrifuged for 10 minutes at 3,000 g. The supernatant (organic phase) was transferred into 15 mL plastic vials to which Na_2_SO_4_.anhydrate had been added. Samples were analyzed using an Agilent Technology 6890 Gas Chromatograph with auto samplers and 5973 Mass Selective Detection and Chemostation Data System (Agilent Technologies, Singapore). Samples (5 μl) were directly injected into the gas chromatograph equipped with an HP-Innowax capillary column (30 m length; 0.25-mm internal diameter, 0.25 μm film thickness; Agilent) using He as gas carrier and a constant flow rate of 0.8 ml/min. The temperature of the injector was kept at 230°C, and the split ratio was 8:1. Chromatographic conditions were as follows: initial oven temperature of 80°C, increase of 8°C/min to 220°C, 12 min at 220°C, and a ramp of 20°C/min up to 230°C to clean the column. In the MS detector, the electron impact energy was set at 70 eV. Data were evaluated with MassHunter software (Agilent Technologies).

### DNA isolation and sequencing of the V3-V4 region of the 16S rRNA gene

DNA isolation and sequencing of barcoded amplicons of the V3-V4 region of the 16S rRNA gene were essentially performed as described before [25] according to established protocols provided by Illumina (Illumina, Eindhoven, the Netherlands). In brief, barcoded amplicons from the V3-V4 region of 16S rRNA genes were generated using a 2-step PCR. In the first step, 10–25 ng genomic DNA was used as template for the first PCR with a total volume of 50 μl using the 341F (5’-CCTACGGGNGGCWGCAG-3’) and 785R (5’-GACTACHVGGGTATCTAATCC-3’) primers appended with Illumina adaptor sequences. PCR products were purified (QIAquick PCR Purification Kit) and the size of the PCR products was checked on a Fragment analyzer (Advanced Analytical, Ankeny, US) and quantified by fluorometric analysis (Qubit™ dsDNA HS Assay Kit). Purified PCR products were used for the second PCR in combination with sample-specific barcoded primers (Nextera XT index kit, Illumina, San Diego, CA, USA). Subsequently, PCR products were purified, checked on a Fragment analyzer and quantified, followed by equimolar multiplexing, and sequencing on an Illumina MiSeq with the paired-end (2x) 300 bp protocol. The sequencing run was analyzed with the Illumina CASAVA pipeline (v1.8.3) with demultiplexing based on sample-specific barcodes. QIIME2 software was used for microbial analyses [26]. Reads were imported and quality filtered and dereplicated with the q2-data2 plugin. Subsequently, the dada2 plugin was used with paired-end reads with truncation of the primer sequences and trimming of the reads. The resulting data were used in the q2-phylogeny plugin to generate a tree for phylogenetic diversity analyses. The sequences were classified using Greengenes (version 13.8) as a reference 16S rRNA gene database. Alpha-diversity (Shannon index, Observed OTUs, evenness and Faith’s PD) and β-diversity analyses (weighted and unweighted UniFrac, Bray-Curtis dissimilarity and Jaccard similarity) were performed with the q2-diversity plugin and visualized in Emporer.

### Statistical analyses

Differences between groups in anthropometric values were evaluated with a T-test. Associations between Amplified Sequence Variants (ASVs)/taxa and different categorical variables, such as stunted/normal nutritional status, gender, sampling site, or age-group, were investigated using the non-parametric Kruskal Wallis test. After the Kruskal–Wallis test, the Benjamini–Hochberg false discovery rate (FDR; a method of conceptualizing the rate of type I errors in null hypothesis testing when conducting multiple comparisons) was applied to correct for multiple comparisons. The non-parametric Spearman's rank-order correlations were obtained between ASVs/taxa and continuous variables, such as age, height and weight. All of these calculations were performed by using the software package R (3.5.3) (R Core Team, http://www.R-project.org/) in RStudio. *Q*-values (adjusted *p*-values after FDR) were considered significantly different at a strict cut-off of *q* < 0.10. Permutational multivariate analysis of variance (PERMANOVA; [27]) was performed to test the significance of difference in β-diversity measures (weighted and unweighted UniFrac, Bray-Curtis dissimilarity and Jaccard similarity) between normal and stunted children in QIIME2.

## Results and discussion

### Characteristics and macronutrient intake of the children with normal nutritional status and stunted Indonesian children 3–5 years of age

**Fig 1:**
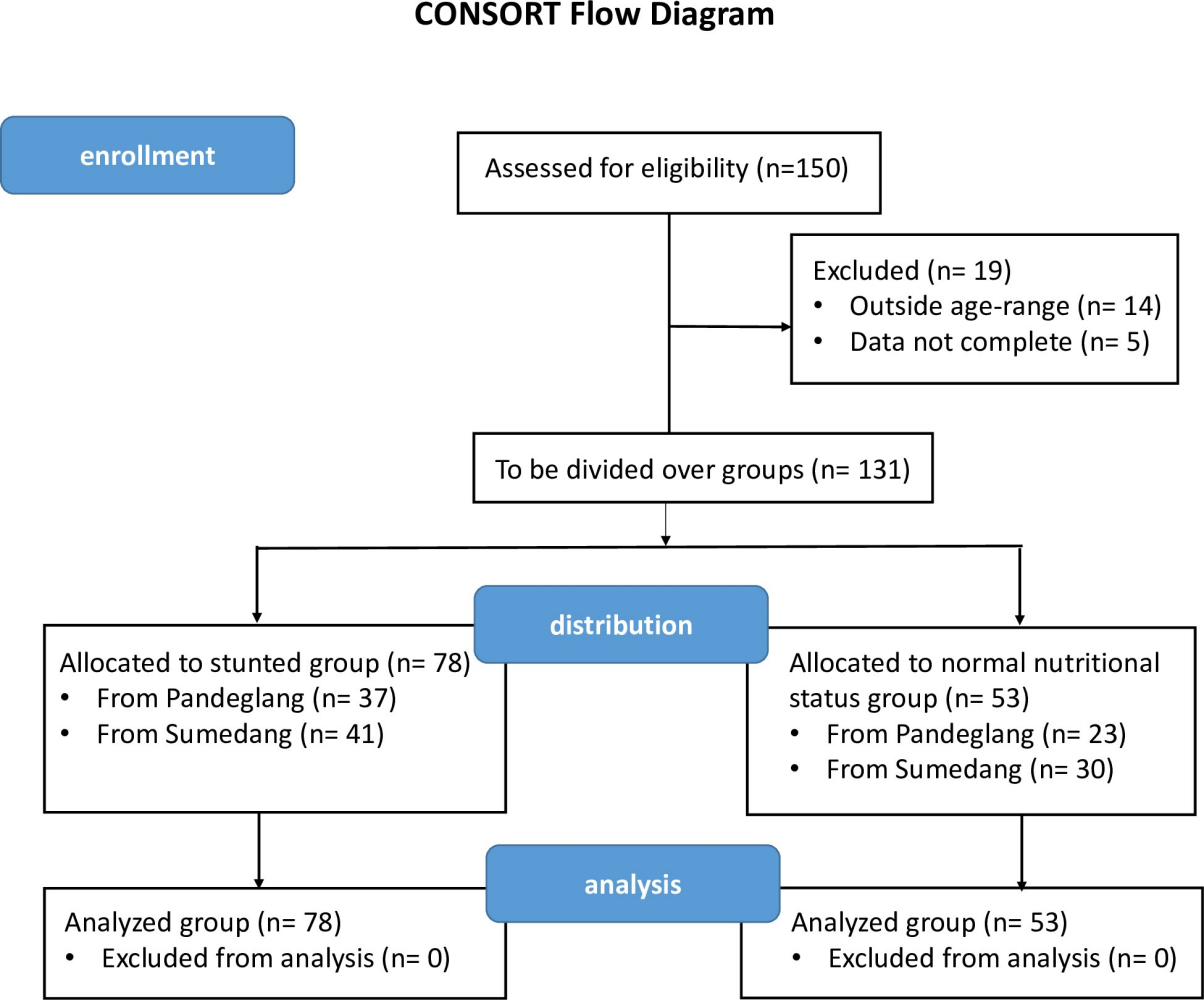
CONSORT flow diagram of the cross-sectional study.

**Fig 2:**
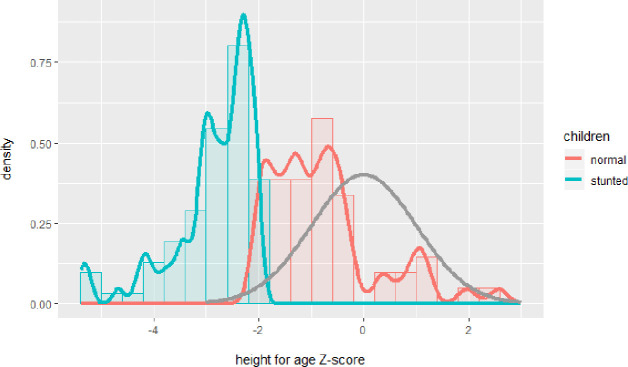
Height for age Z-score of the Indonesian children. Stunted: green; normal: red; plotted against the WHO reference dataset: grey.

This was a cross-sectional study, without intervention. Fig 1 shows the CONSORT flow chart of the study. Table 1 shows the anthropometric parameters of the two groups of children of 3–5 years segregated based on their height-for-age Z-score. Fig 2 shows the distribution of the height-for-age Z-scores of stunted children (Fig 2, red, *n* = 78) and children with normal nutritional status (Fig 2, green; *n* = 53), plotted against the WHO reference dataset (Fig 2, grey). Stunting is defined as Z-score < -2, and severe stunting as Z-score < -3. Some of the children had a Z-score lower than -4 (*n* = 8), with the lowest score being -5.39. From the distribution of the children considered to be of normal nutritional status, it is clear that most of these (*n* = 43 of 53) are to the left of the zero-line in the WHO reference data-set, and therefore on average still shorter than the average height-for-age in the WHO-dataset.

**Table 1:** Anthropometric data, macronutrient- and energy-intake, and fecal SCFA and BCFA concentrations of the groups.

	normal	stunted	*p or q*-value ^†^
Gender (M/F)	36/17	37/41	
Age (month) ^#^	48.57 ± 8.68	49.54 ± 8.54	0.68
Birth weight (kg) ^#^	3.25 ± 0.44	3.04 ± 0.46	0.11
Weight (kg) ^#^	14.80 ± 3.78	12.58 ± 1.34	**0.013**
Height/length (cm) ^#^	99.88 ± 7.48	91.78 ± 4.57	9.4 * 10^**−5**^
BMI (kg*m^-2^) ^#^	14.81 ± 2.21	15.20 ± 2.02	0.30
Energy (kcal) ^#^	1,160 ± 430	1,011 ± 490	0.18
Carbohydrate (g) ^#^	165.15 ± 70.79	144.59 ± 472.07	0.26
Fat (g) ^#^	38.19 ± 18.70	32.24 ± 18.47	0.17
Protein (g) ^#^	34.91 ± 15.00	34.59 ± 28.77	0.24
Dietary fiber (g) ^#^	5.01 ± 3.13	4.30 ± 2.28	0.46
Acetate (mM) ^#^	3.57 ± 3.87	6.28 ± 6.42	0.195
Propionate (mM) ^#^	2.36 ± 4.73	4.27 ± 10.11	0.63
*n*-Butyrate (mM) ^#^	1.67 ± 2.01	2.62 ± 3.47	0.54
*n*-Valerate (mM) ^#^	0.38 ± 0.44	0.87 ± 1.82	0.13
*iso*-Butyrate (mM) ^#^	0.13 ± 0.19	0.37 ± 0.76	0.24
*iso*-Valerate (mM) ^#^	0.25 ± 0.63	0.70 ± 1.56	0.24
SCFA (sum of acetate, propionate, *n*-butyrate and *n*-valerate) ^#^	7.97 ± 9.72	14.03 ± 17.48	0.13
BCFA (sum of *iso*-butyrate and *iso*-valerate) ^#^	0.39 ± 0.81	1.07 ± 1.94	0.24

^†^ significant values in bold; *q*-values underlined.

^#^ average ± SD

The intake of all macronutrients (carbohydrates, fat, protein and dietary fiber) and energy was lower in stunted children compared to children with normal nutritional status, with p < 0.05 for fat- and energy-intake, although this was no longer significant after correcting for multiple comparisons (Table 1). Nevertheless, for fat- and energy-intake the *q*-value was still lower than 0.2, which is usually taken as the cut-off for significance for FDR. We however, prefer to keep a stricter cut-off and consider the difference in fat- and energy-intake a trend towards significance.

### Energy excretion in the form of fecal SCFA in children with normal nutritional status and stunted children

Apart from a reduced energy- and macronutrient-intake, we also observed a higher concentration of SCFA (acetate, propionate, *n*-butyrate and *n*-valerate) and branched-chain fatty acids (BCFA; *iso*-butyrate and *iso*-valerate) in collected stool samples of stunted children. There was a trend for significance after correcting for multiple comparisons for acetate, valerate and total SCFA (sum of acetate, propionate, butyrate and valerate) (Table 1; *q* < 0.2 for all three). Unfortunately, total stool output was not recorded, but assuming similar output in both groups, this would mean energy-loss in the form of SCFA and BCFA in stunted children compared to children with normal nutritional status. Assuming approximately 25 g stool output per day (which is considered the average for children in the age-range of 1–4 years [28]), this would amount on average to 63 kcal/day energy loss, on top of the on average 150 kcal/day lower energy-intake. This would mean a 1 kilogram difference in bodyweight in about a month (36 days). Of course, this is currently primarily speculation, and situations for the children will change on a day-to-day basis. Moreover, also potential differences in energy-expenditure should be taken into consideration, but it is interesting that the differences observed here could explain some of the observed differences in weight and ultimately height (Table 1).

### Gut microbiota differences between children with normal nutritional status and stunted Indonesian children 3–5 years of age

The gut microbiota of 78 stunted children (S) of 3–5 years from Pandeglang (P) and Sumedang (Su) were compared to 53 children of 3–5 years with normal nutritional status (N) of the same age. Sample SSu20 did not contain any sequence data and was excluded from further analyses.

As observed in numerous other studies, the major phyla were Bacteroidetes and Firmicutes (Table 2). This was followed by Proteobacteria. Both Actinobacteria and Verrucomicrobia were present at 0.5% - 1%. Ten other phyla were present only in some samples and were grouped under “Other” in Table 2.

**Table 2:** Gut microbiota composition at the phylum level.

Phylum	N	S	*p*-value
	*n* = 53	*n* = 77	
Actinobacteria	0.75%	1.12%	
Bacteroidetes	51.29%	44.39%	2.55*10^−4^
Firmicutes	39.81%	45.71%	5.89*10^−4^
Proteobacteria	6.21%	6.40%	
Verrucomicrobia	0.57%	0.47%	
Other	1.36%	1.91%	

The relative abundances (RA) of Bacteroidetes (51.3% for children with normal nutritional status (N) vs. 44.4% for stunted children (S); *p*-value 2.55*10^−4^) and Firmicutes (39.8% vs. 45.7%, respectively; *p*-value 5.89*10^−4^) were significantly different between the groups. The drop in RA in Bacteroidetes in the stunted children was accompanied by an equal raise in RA of Firmicutes, and in both groups these two phyla made up 90–91% of the phyla. There were no significant changes in the other phyla.

At the family level, only two families showed a significant difference (*q* < 0.1) in RA, namely *Ruminococcaceae* and an uncharacterized (uncultured) family of *Mollicutes* (*q* = 0.043 for both). Both families were higher in the stunted children. None of the other families were significantly different between the stunted children and children with normal nutritional status.

**Fig 3:**
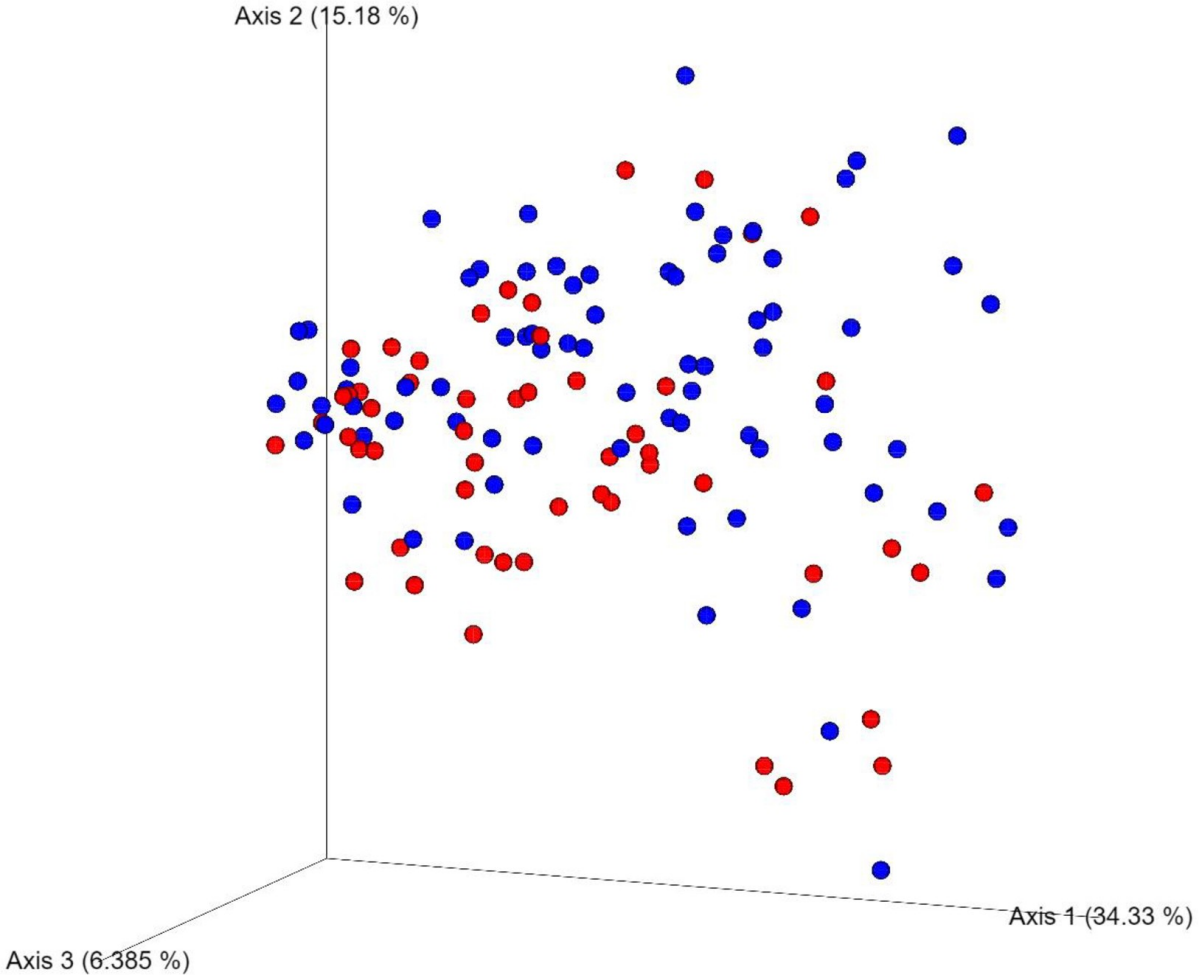
Weighted UniFrac for the normal nutritional status (red) and stunted (blue) children.

Significant differences were observed in β-diversity between the two groups (Fig 3 for weighted UniFrac, PERMANOVA *p*-value = 0.002; R^2^ = 0.031; S1 Fig for unweighted UniFrac [*p* = 0.017; R^2^ = 0.018], Bray-Curtis dissimilarity [*p* = 0.015; R^2^ = 0.012] and Jaccard similarity plots [*p* = 0.026; R^2^ = 0.010]). There were also significant differences in the 4 calculated α-diversity measures: Shannon index, observed OTUs, Faith phylogenetic diversity (PD) and evenness (S1D Fig; all *q* < 0.1). The four metrics all showed a higher α-diversity in the stunted children. Increased α-diversity in adults is commonly considered to be correlating with a healthy/healthier status. However, other parameters than α-diversity seem to be better predictors for this [29], although it is unclear whether that is also the case for children, as this has been established only in adults. Also, how α-diversity in children relates to health is as yet unclear.

**Fig 4:**
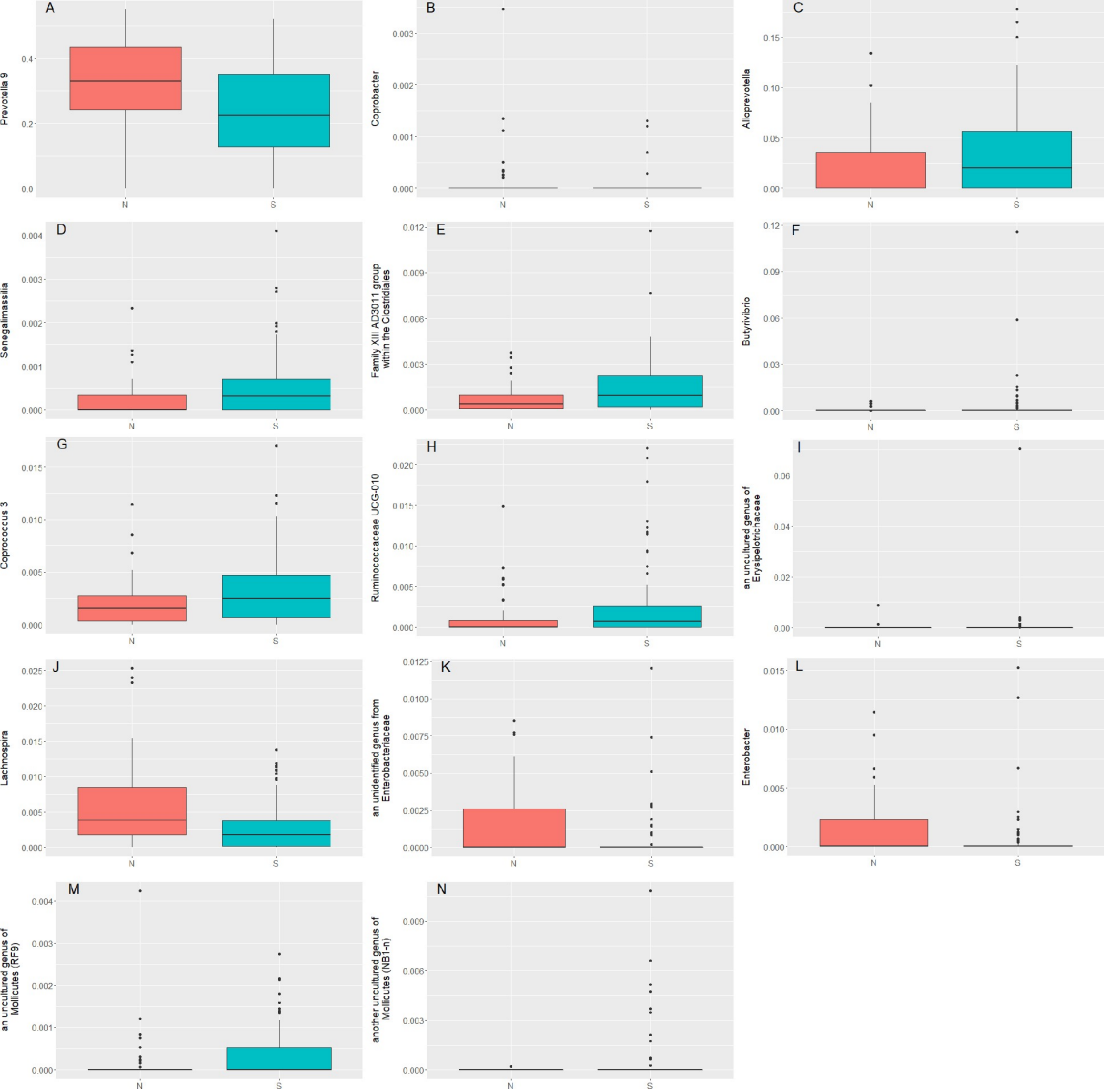
The 14 taxa at genus level that were different between normal nutritional status (N; red) and stunted (S; green) children. A. *Prevotella* 9; B. *Coprobacter*; C. *Alloprevotella*; D. *Senegalimassilia*; E. Family XIII AD3011 group within the Clostridiales, F. *Butyrivibrio*; G. *Coprococcus* 3, H. *Ruminococcaceae* UCG-010; I. an uncultured genus of *Erysipelotrichaceae*; J. *Lachnospira*; K. an unidentified genus from *Enterobacteriaceae*; L. *Enterobacter*, M. an uncultured genus of *Mollicutes* (RF9); N. another uncultured genus of *Mollicutes* (NB1-n).

There were 14 individual taxa that showed significant differences (*q*-value < 0.1) between the groups in a non-parametric Kruskal Wallis test corrected for FDR (Fig 4). Within the Bacteroidetes phylum, the taxon *Prevotella* 9, which was by far the most abundant ASV in the whole group (on average 26.3% RA; followed by *Bacteroides* with 8.7%) was significantly higher in the children with normal nutritional status (N; 30.5%) than in the stunted children (S; 23.5%; *q*-value 0.059; Fig 4A). Two other taxa within the Bacteroidetes phylum were also significantly different: *Coprobacter* (Fig 4B; *q*-value 0.099; lower in stunted children) and *Alloprevotella* (Fig 4C; *q*-value 0.042; higher in stunted children). Within the Actinobacteria phylum the genus *Senegalimassilia* was significantly higher in stunted children (Fig 4D; *q*-value 0.052).

Within the phylum Firmicutes several taxa were different between the two groups. The taxa “Family XIII AD3011 group” within the Clostridiales (Fig 4E; *q*-value 0.079), *Butyrivibrio* (Fig 4F; *q*-value 0.079), *Coprococcus* 3 (Fig 4G; *q*-value 0.068), *Ruminococcaceae* UCG-010 (Fig 4H; *q*-value 0.029) and an uncultured genus of Erysipelotrichaceae (Fig 4I; *q*-value 0.005) were higher in stunted children, while *Lachnospira* was higher in normal children (Fig 4J; *q*-value 0.015). Within the Proteobacteria (specifically γ-Proteobacteria) an unidentified genus from *Enterobacteriaceae* (Fig 4K; *q*-value 0.043) and *Enterobacter* (Fig 4L; *q*-value 0.079) were lower in stunted children. Within the Tenericutes phylum, specifically the order Mollicutes, 2 uncharacterized taxa were higher in stunted children; (Fig 4M and 4N; *q*-value 0.068 and 0.022, respectively). Some of these 14 taxa were present at very low abundance, and only present in a few children and their biological relevance is questionable. E.g. *Coprobacter* was mostly present in normal children (Fig 4B; *n =* 10 and *n =* 4 for normal and stunted, respectively), whereas *Butyrivibrio* was mostly present in stunted children (Fig 4F; *n =* 6 and *n =* 23 for normal and stunted, respectively). This was also the case for the uncultured genus of *Erysipelotrichaceae* (Fig 4I; *n =* 2 and *n =* 22 for normal and stunted) and one of the uncharacterized taxa within the Mollicutes order (Fig 4M; *n =* 1 and *n =* 12 for normal and stunted). *Bacteroides* (10.4% RA and 7.6% RA for normal and stunted, respectivelty; difference non-significant [NS]) and *Faecalibacterium* (7.7% RA and 7.4% RA for normal and stunted, respectively; NS) were the only genera present in all children, while *Prevotella* 9, that was the genus with the highest relative abundance, was missing from one stunted child.

Since it is known that the gut microbiota is rather dynamic at young age, we studied the correlation between taxa and age. Five genera were negatively correlated with age, none positively (strict cut-off of *q*-value of 0.01; Table 3). The scatter-plots of the 5 taxa are shown in S2 Fig.

**Table 3:** Taxa significantly different with age.

Taxa	*q*-value	rho
*Akkermansia*	5.01*10^−5^	-0.383
*Pyramidobacter*	0.014	-0.271
*Alistipes*	0.017	-0.265
*Comamonas*	0.055	-0.233
*Providencia*	0.095	-0.216

Several taxa correlated with height, weight and BMI (Table 4; S3 Fig for scatterplots). Only one of the 14 taxa that were different between normal and stunted children, namely *Prevotella* 9, correlated with height, even though height was the discriminating factor between the two groups. It also was the only taxon that positively correlated with weight (Table 4).

**Table 4:** Spearman correlations between taxa and weight, height and BMI.

height/taxa	*q*-value	rho
*Ruminococcaceae* UCG-014	0.01	-0.282
uncultured genus of Mollicutes (RF9)	0.01	-0.28
*Leuconostoc*	0.02	-0.267
*Prevotella* 9	0.05	0.238
uncultured genus of Gastranaerophilales	0.05	-0.236
*Desulfovibrio*	0.06	-0.232
*Intestinimonas*	0.08	-0.220
*Caproiciproducens*	0.09	-0.216
**weight/taxa**	***q*-value**	**rho**
*Prevotella* 9	0.01	0.279
**bmi/taxa**	***q*-value**	**rho**
*Leuconostoc*	0.05	0.238
*Alloprevotella*	0.05	0.237
*Bacteroides*	0.05	-0.237
*Catenibacterium*	0.09	0.220

There were two taxa significantly different between gender: *Paraprevotella* (*q* = 0.06) and *Comamonas* (*q* = 0.07) were both higher in girls (S4 Fig), but both were present at low abundance (below 0.18% RA) and present in a limited number of children (*Paraprevotella n =* 17 and *n =* 21, and *Comamonas n =* 11 and *n =* 14 in normal and stunted children, respectively) and thus the biological relevance of this is questionable. There was no difference between normal and stunted children (S4 Fig).

**Fig 5:**
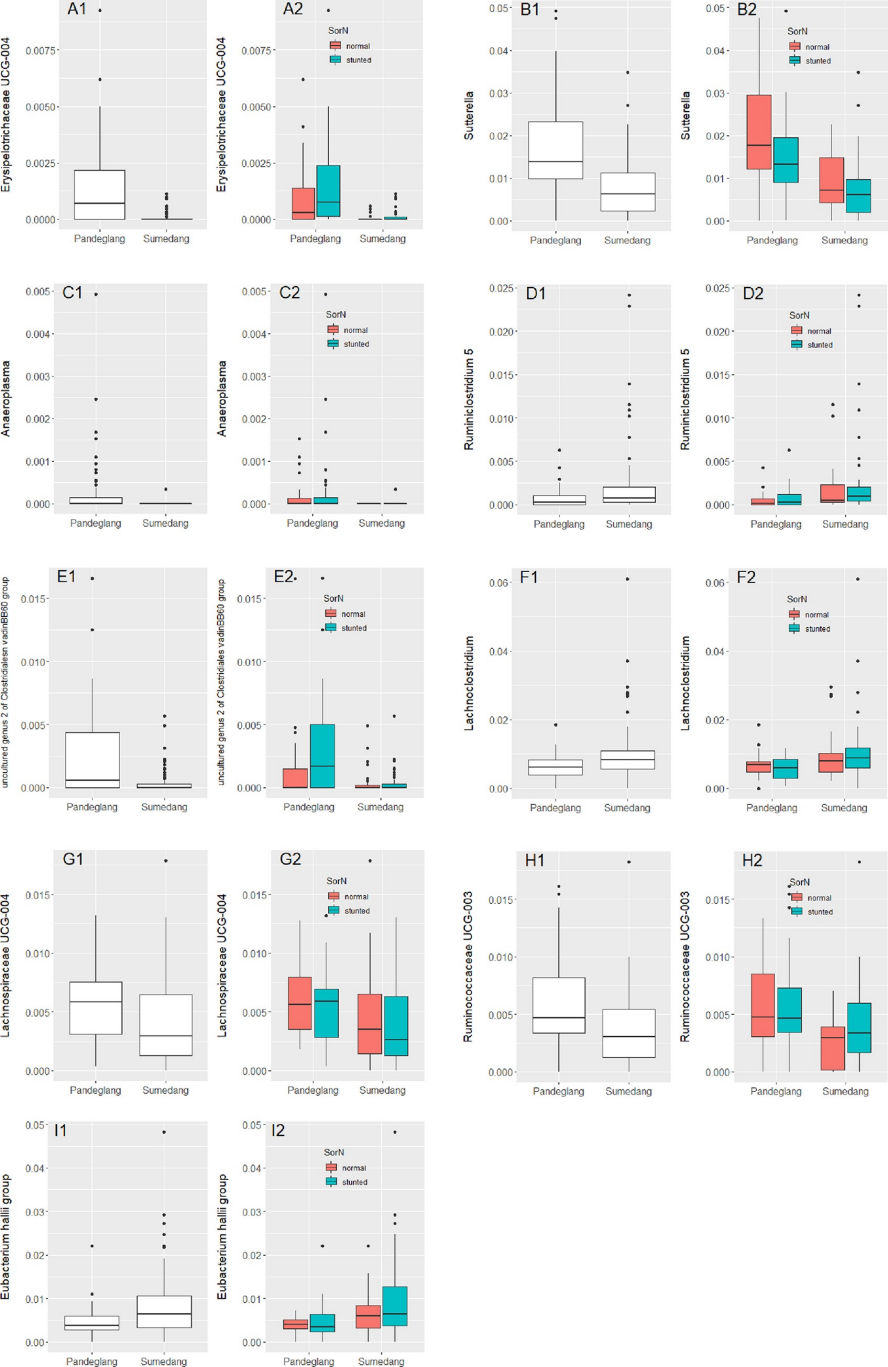
Nine of the 20 taxa at genus level that were different between sampling site (P and Su) (A1-I1) and on top of that different between normal nutritional status (N) and stunted (S) children (A2-I2). A. Erysipelotrichaceae UCG-004; B. Sutterella; C. Anaeroplasma; D. Ruminiclostridium 5; E. uncultured genus 2 of Clostridiales vadinBB60 group; F. Lachnoclostridium; G. Lachnospiraceae UCG-004; H. Ruminococcaceae UCG-003; I. Eubacterium hallii group.

There were 40 taxa that were significantly different between sampling sites (Pandeglang [P] and Sumedang [Su], *q*-value < 0.1; S1 Table). Of these, 20 showed also a significant difference between normal and stunted children when split by sampling site. Of the 40 taxa, 11 had low prevalence and/or abundances. For the other 9 that showed significant difference between children of normal nutritional status and stunted children (S1 Table), the difference in sampling sites is indicated in Fig 5.

For some of the 9 taxa, observations at one sampling site are mirrored by the other sampling site. E.g., for *Erysipelotrichaceae* UCG-004 (Fig 5A), *Anaeroplasma* (Fig 5C), *Ruminiclostridium* 5 (Fig 5D) and the uncultured genus 2 of Clostridiales vadinBB60 group (Fig 5E) the RA was higher in stunted children than in normal children. On the other hand, for *Sutterella* (Fig 5B) the RA was higher in normal children than in stunted at both sites. However, for *Lachnoclostridium* (Fig 5F), *Lachnospiraceae* UCG-004 (Fig 5G), *Ruminococcaceae* UCG-003 (Fig 5H) and *Eubacterium hallii* group (Fig 5I), the RA was either higher in normal or in stunted children, depending on the sampling site.

Most of the 14 taxa that were significantly different between stunted children and children of normal nutritional status (Fig 4) were present at low abundance (from 0.008% RA for *Coprobacter* up to an average RA of 3.0% for *Alloprevotella*, although there was a tremendous inter-individual variation, with one of the stunted children having 17.8% RA of *Alloprevotella*). Although it cannot be excluded that these low abundant taxa play a role in stunting, it is more likely that *Prevotella* 9, which was very abundant in the children (on average 27%; range 0–55.1%) and which was lower in the stunted children (average 23.5% vs. 30.5% for the children with normal nutritional status), plays a role in stunting. *Prevotella* has been associated with long-term dietary fibre intake [30], and our nutritional intake data showed that dietary fiber intake (along with all other macronutrients and energy-intake) was lower in stunted children. *Prevotella* 9 however did not correlate with dietary fibre-intake. Only three taxa did: *Erysipelotrichaceae* UCG-004, an uncharacterized genus of the *Erysipelotrichaceae* family, and *Lachnospiraceae* UCG-008 significantly (*q* < 0.1) negatively correlated with dietary fiber intake. The gut microbiota ferments dietary fibres into SCFA, which are, amongst others, used by the host as energy source in e.g. the colonic epithelium [31], liver, muscle [13, 15, 16], and brain [32]. In this manner, *Prevotella* may contribute to additional energy extraction from the diet in the form of SCFA, which would be helpful for stunted children. It was therefore striking to observe that the concentrations of all individual SCFA (and hence also the sum of the SCFA) were higher in feces of stunted children, indicating increased energy-loss though fecal excretion. Unfortunately, we did not record total fecal output, so we cannot exclude a lower fecal output in stunted children, which would negate a higher concentration in feces, but if fecal output was similar in both groups, energy-loss in the form of SCFA was higher in the stunted children. It has been estimated that approximately 5–10% of daily energy needs could be met by the SCFA produced by the gut microbiota [33–35]. In an interesting study, it was found, based on whole-genome metabolic modelling of 68 gut microbes, that there may be reduced production of certain amino acids in stunted children compared to children with normal nutritional status from the same communities [12]. Indeed, plasma metabolic profiling revealed that stunted children had reduced plasma levels of essential amino acids compared to healthy controls [12]. Moreover, the two short-chain fatty acids propionate and butyrate, and the two TCA cycle intermediates pyruvate and malate were found to be significantly higher in the plasma of stunted children compared to the healthy group [12]. Unfortunately, no link (correlation) was made with the gut microbiota composition, although the data were available [18]. These results seem to be inconsistent with our data, where we show increased fecal SCFA (and BCFA) concentrations in stunted children.

Unlike earlier observations in the microbiota of stunted children in Africa [19] and severe acute malnutrition children in Bangladesh [18], in the current study members of the Enterobacteriaceae were not higher in stunted children (1.88% RA at family level) than in children with normal nutritional status (2.14% RA). In fact, two taxa within the Enterobacteriaceae, namely an unidentified genus from Enterobacteriaceae and *Enterobacter* were significantly lower in stunted children than in normal children (Fig 4K and 4L, *q* = 0.044 and 0.079 respectively). Vonaesch *et al*. [19] particularly found increased *E*.*coli*/*Shigella* in stunted children. In our study this taxon was slightly lower (but non-significant) in stunted children (1.34% RA) than in normal children (1.5% RA). In the African dataset, *E*.*coli*/*Shigella* was prevalent in 79% (0.027% average RA, Central African Republic) to 92% (0.64% average RA, Madagascar) of the individuals. In our Indonesian dataset, *E*.*coli*/*Shigella* was present in 48 out of 53 (83%) normal children and 71 out of 78 (91%) stunted children. These authors also found *Campylobacter* to be higher in stunted children, whereas the RA of Campylobacter was equal in our study (0.71% vs 0.79% RA for stunted and normal children, respectively). In the African dataset, *Campylobacter* was found in all children at an average RA of 0.21% (Central African Republic) - 0.35% (Madagascar). In our dataset, *Campylobacter* was only present in 11 out of 53 normal children and 16 out of 78 stunted children. In that study α-diversity was not different between stunted and healthy children [19]. Also Dinh *et al*. [36] observed an increase in taxa of the Campylobacterales order in stunted children in India up to two years of age, on top of an increase in *Desulfovibrio* (no information on the RA available). In our study, the difference between normal and stunted children for *Desulfovibrio* did not reach statistical significance after FDR correction (q = 0.38; 0.27% vs 0.38% for normal and stunted children, respectively). The difference may be due to the different age groups tested (from 0–2 years in Dinh *et al*.; from 3–5 years in our study). The microbiota of very young children is still quite dynamic, and only stabilizes around 3 years [37], which is why we started sampling at this age. The dynamics of the microbiota is corroborated by Dinh *et al*., who observed that the Bacteroidetes phylum was higher in stunted compared to control children at 12 months of age, but no longer significantly different at 2 years of age [36].

Herpertz‑Dahlmann *et al*. [14] reviewed the effect of anorexia nervosa on gut microbiota. They observed that starvation-induced changes in the gut microbiome are correlated to intestinal barrier dysfunction. A disturbed gut barrier function or leaky gut was also found in other disorders associated with malnourishment and in volunteered fasting subjects [14]. Although there are very few human studies in anorexic patients, one study found a ~3.5 increased amount of *Methanobrevibacter smithii*, as measured by q-PCR [38]. Also our stunted children could be considered starved, and although *Methanobrevibacter* was about 4-fold higher in stunted children compared to normal children (RA 0.076% and 0.019%, respectively), this was not significant.

Our data on *Prevotella* 9 being the most prevalent taxon in the Indonesian children is entirely in line with data from the Asian Microbiome project [21], which showed high *Prevotella* in school-aged children in the city of Yogyakarta and on Bali (in addition to the city of Khon Kaen in Thailand), compared to other Asian countries [21]. Some of the differences in microbial communities in the included Asian countries were traced back to diet. The data by Nakayama *et al*. [21] show that the gut microbiota (of school-aged children) is very region-specific, explaining some of the discrepancies between our results and some of the studies discussed above.

A limitation of the current study is that it is not longitudinal. The microbiome may play a significant role in limiting growth of children, because increased intestinal permeability and exposure to infections both disturb intestinal functioning as well as normal growth. Moreover, changes in microbiome-induced gut barrier function may influence micronutrient bioavailability and metabolic processes [11], but little is known about changes in the microbiome during periods of undernutrition. Thus, improving the understanding of how the microbiome changes during nutrient deprivation is of great scientific and public health importance. Especially in low-income countries the double burden (economically and hygienically) leads to poor child growth. Thus, research is warranted to advance the knowledge of the long-term role of the microbiome on the growth and health of children exposed to undernutrition and infections, to prevent a vicious circle from occurring: poor growth as a consequences of undernutrition leads to underdevelopment of the brain and disturbed cognitive development, which leads to lower productivity as well as lower chances of economic success. In turn, lower productivity and lower economic success will again result in undernutrition and poor growth, also in subsequent generations.

## Conclusion

In conclusion, this is one of a few studies that looked at the microbiota composition in stunted children and compared it to children with normal nutritional status of the same age. *Prevotella* 9 was the most abundant in the Indonesian children, and was significantly lower in the stunted children compared to the children with normal nutritional status, indicating lower dietary fibre intake, which was corroborated by the nutritional intake data. Hence, increasing the proportion of *Prevotella* in stunted children (e.g. through fruit and vegetable intake) may be of benefit. Whether this would prevent the occurrence of stunting or even has the potential to revert it, remains to be seen in follow up research that is planned at multiple regions in Indonesia. It may be that one or more of the other taxa that were different between the two groups also play a role. Future research, preferably in a longitudinal study should clarify this.

## Supporting information

S1 Checklist TREND statement checklist—PONE-D-20-26845; non-randomized, cross-sectional study. (PDF)

S1 Table Taxa that were different between sampling sites (Pandeglang [P] and Sumedang [Su]) and/or different between normal (N) or stunted (S) children when split up by sampling site. (DOCX)

S1 Fig A. Unweighted UniFrac; B. Bray-Curtis, and C. Jaccard PCoA plots for the normal nutritional status (red) and stunted (blue) children. D: alpha-diversity indices Shannon index, observed OTUs, Faith’s phylogenetic diversity (PD), and evenness. All metrics *q* < 0.06.
(DOCX)

S2 Fig Scatterplots of the five taxa that significantly negatively correlated with age. A. *Akkermansia*, correlation coefficient -0.383; B. *Pyramidobacter*, correlation coefficient -0.271; C. *Alistipes*; correlation coefficient -0.265; D. *Comamonas*, correlation coefficient -0.233; E. *Providencia*, correlation coefficient -0.216.
(DOCX)

S3 Fig Scatterplots of the taxa that are significantly correlated with height (A-H), weight (I) and BMI (J-M). A. *Ruminococcaceae* UCG-014, correlation coefficient -0.282; B. uncultured genus of Mollicutes(RF9), correlation coefficient -0.280; C. *Leuconostoc*; correlation coefficient -0.267; D. *Prevotella* 9, correlation coefficient +0.238; E. uncultured genus of Gastranaerophilales, correlation coefficient -0.236; F. *Desulfovibrio*, correlation coefficient -0.232; G. *Intestinimonas*, correlation coefficient -0.220; H. *Caproiciproducens*, correlation coefficient -0.216; I. *Prevotella* 9, correlation coefficient +0.279; J. *Leuconostoc*, correlation coefficient +0.238; K. *Alloprevotella*, correlation coefficient +0.237; L. *Bacteroides*, correlation coefficient -0.237; M., correlation coefficient -0.21; N. *Catenibacterium*, correlation coefficient +0.220.
(DOCX)

S4 Fig Boxplots of the 2 taxa that are significantly correlated with gender. (DOCX)

S1 Study (PDF)

S2 Study (PDF)

## References

[1] World Health Organization. WHO child growth standards and the identification of severe acute malnutrition in infants and children—A Joint Statement by the World Health Organization and the United Nations Children’s Fund. 2009; available at: https://www.who.int/nutrition/publications/severemalnutrition/9789241598163/en/. 24809116

[2] Black RE, Victora CG, Walker SP, Bhutta ZA, Christian P, de Onis M, et al Maternal and child undernutrition and overweight in low-income and middle-income countries. Lancet. 2013;382(9890):427–51. Epub 2013/06/12. 10.1016/S0140-6736(13)60937-X . 23746772

[3] Prendergast AJ, Humphrey JH. The stunting syndrome in developing countries. Paediatr Int Child Health. 2014;34(4):250–65. Epub 2014/10/14. 10.1179/2046905514Y.0000000158 25310000 PMC4232245

[4] United Nations Children’s Fund (UNICEF), World Health Organization, International Bank for Reconstruction and Development/The World Bank. Levels and trends in child malnutrition: Key Findings of the 2020 Edition of the Joint Child Malnutrition Estimates. Geneva: World Health Organization; 2020.

[5] United Nations Children’s Fund. Tracking Progress on Child and Maternal Nutrition—A survival and development priority. New York: 2009. Available at: https://www.unicef.org/publications/files/Tracking_Progress_on_Child_and_Maternal_Nutrition_EN_110309.pdf.

[6] World Health Organization. Global targets to improve maternal, infant and young child nutrition for 2025. 2018. Available at https://www.who.int/nutrition/topics/nutrition_globaltargets2025/en/.

[7] Scheffler C, Hermanussen M, Bogin B, Liana DS, Taolin F, Cempaka P, et al Stunting is not a synonym of malnutrition. Eur J Clin Nutr. 2020;74(3):377–86. Epub 2019/05/31. 10.1038/s41430-019-0439-4 . 31142828

[8] Central Bureau of Statistics- Ministry of Health Indonesia. Report on Integration of Economic Social Survey (Susenas) Implementation, March 2019 and Nutritional Status of Children Younger than Five Study in Indonesia (SSGBI). Jakarta: 2019. (in Indonesian).

[9] Ministry of Healthy Indonesia. Main results of 2018’s Indonesia Basic Health Survey. 2018. Available at: http://kesmas.kemkes.go.id/assets/upload/dir_519d41d8cd98f00/files/Hasil-riskesdas-2018_1274.pdf (in Indonesian).

[10] de Onis M, Branca F. Childhood stunting: a global perspective. Matern Child Nutr. 2016;12 Suppl 1:12–26. Epub 2016/05/18. 10.1111/mcn.12231 27187907 PMC5084763

[11] Hoffman DJ, Campos-Ponce M, Taddei CR, Doak CM. Microbiome, growth retardation and metabolism: are they related? Ann Hum Biol. 2017;44(3):201–7. Epub 2016/12/09. 10.1080/03014460.2016.1267261 . 27927018

[12] Kumar M, Ji B, Babaei P, Das P, Lappa D, Ramakrishnan G, et al Gut microbiota dysbiosis is associated with malnutrition and reduced plasma amino acid levels: Lessons from genome-scale metabolic modeling. Metab Eng. 2018;49:128–42. Epub 2018/08/04. 10.1016/j.ymben.2018.07.018 30075203 PMC6871511

[13] Guida S, Venema K. Gut microbiota and obesity: Involvement of the adipose tissue. Journal of Functional Foods. 2015;14:407–23.

[14] Herpertz-Dahlmann B, Seitz J, Baines J. Food matters: how the microbiome and gut-brain interaction might impact the development and course of anorexia nervosa. Eur Child Adolesc Psychiatry. 2017;26(9):1031–41. Epub 2017/02/02. 10.1007/s00787-017-0945-7 28144744 PMC5591351

[15] Canfora EE, van der Beek CM, Jocken JWE, Goossens GH, Holst JJ, Olde Damink SWM, et al Colonic infusions of short-chain fatty acid mixtures promote energy metabolism in overweight/obese men: a randomized crossover trial. Sci Rep. 2017;7(1):2360 Epub 2017/05/26. 10.1038/s41598-017-02546-x 28539646 PMC5443817

[16] Canfora EE, Meex RCR, Venema K, Blaak EE. Gut microbial metabolites in obesity, NAFLD and T2DM. Nat Rev Endocrinol. 2019;15(5):261–73. Epub 2019/01/24. 10.1038/s41574-019-0156-z . 30670819

[17] Vonaesch P, Randremanana R, Gody JC, Collard JM, Giles-Vernick T, Doria M, et al Identifying the etiology and pathophysiology underlying stunting and environmental enteropathy: study protocol of the AFRIBIOTA project. BMC Pediatr. 2018;18(1):236 Epub 2018/07/22. 10.1186/s12887-018-1189-5 30025542 PMC6053792

[18] Subramanian S, Huq S, Yatsunenko T, Haque R, Mahfuz M, Alam MA, et al Persistent gut microbiota immaturity in malnourished Bangladeshi children. Nature. 2014;510(7505):417–21. Epub 2014/06/05. 10.1038/nature13421 24896187 PMC4189846

[19] Vonaesch P, Morien E, Andrianonimiadana L, Sanke H, Mbecko JR, Huus KE, et al Stunted childhood growth is associated with decompartmentalization of the gastrointestinal tract and overgrowth of oropharyngeal taxa. Proc Natl Acad Sci U S A. 2018;115(36):E8489–E98. Epub 2018/08/22. 10.1073/pnas.1806573115 30126990 PMC6130352

[20] Gehrig JL, Venkatesh S, Chang HW, Hibberd MC, Kung VL, Cheng J, et al Effects of microbiota-directed foods in gnotobiotic animals and undernourished children. Science. 2019;365(6449). Epub 2019/07/13. 10.1126/science.aau4732 31296738 PMC6683325

[21] Nakayama J, Watanabe K, Jiang J, Matsuda K, Chao SH, Haryono P, et al Diversity in gut bacterial community of school-age children in Asia. Sci Rep. 2015;5:8397 Epub 2015/02/24. 10.1038/srep08397 25703686 PMC4336934

[22] Arumugam M, Raes J, Pelletier E, Le Paslier D, Yamada T, Mende DR, et al Enterotypes of the human gut microbiome. Nature. 2011;473(7346):174–80. Epub 2011/04/22. 10.1038/nature09944 21508958 PMC3728647

[23] Kolde R, Franzosa EA, Rahnavard G, Hall AB, Vlamakis H, Stevens C, et al Host genetic variation and its microbiome interactions within the Human Microbiome Project. Genome Med. 2018;10(1):6 Epub 2018/01/31. 10.1186/s13073-018-0515-8 29378630 PMC5789541

[24] Schnorr SL, Candela M, Rampelli S, Centanni M, Consolandi C, Basaglia G, et al Gut microbiome of the Hadza hunter-gatherers. Nat Commun. 2014;5:3654 Epub 2014/04/17. 10.1038/ncomms4654 24736369 PMC3996546

[25] Venema K, Verhoeven J, Verbruggen S, Keller D. Xylo-oligosaccharides from sugarcane show prebiotic potential in a dynamic computer-controlled in vitro model of the adult human large intestine. Benef Microbes. 2020;11(2):191–200. Epub 2020/03/27. 10.3920/BM2019.0159 . 32208927

[26] Bolyen E, Rideout JR, Dillon MR, Bokulich NA, Abnet CC, Al-Ghalith GA, et al Reproducible, interactive, scalable and extensible microbiome data science using QIIME 2. Nat Biotechnol. 2019;37(8):852–7. Epub 2019/07/26. 10.1038/s41587-019-0209-9 31341288 PMC7015180

[27] Anderson MJ. Permutational Multivariate Analysis of Variance (PERMANOVA). In: Balakrishnan N, Colton T, Everitt B, Piegorsch W, Ruggeri F, Teugels JL, editors. Wiley StatsRef: Statistics Reference Online 2017.

[28] Weaver LT, Steiner H. The bowel habit of young children. Arch Dis Child. 1984;59(7):649–52. Epub 1984/07/01. 10.1136/adc.59.7.649 6087745 PMC1628927

[29] Gupta VK, Kim M, Bakshi U, Cunningham KY, Davis JM 3rd, Lazaridis KN, et al A predictive index for health status using species-level gut microbiome profiling. Nat Commun. 2020;11(1):4635 Epub 2020/09/17. 10.1038/s41467-020-18476-8 32934239 PMC7492273

[30] Wu GD, Chen J, Hoffmann C, Bittinger K, Chen YY, Keilbaugh SA, et al Linking long-term dietary patterns with gut microbial enterotypes. Science. 2011;334(6052):105–8. Epub 2011/09/03. 10.1126/science.1208344 21885731 PMC3368382

[31] Roediger WE. Utilization of nutrients by isolated epithelial cells of the rat colon. Gastroenterology. 1982;83(2):424–9. Epub 1982/08/01. . 7084619

[32] Frost G, Sleeth ML, Sahuri-Arisoylu M, Lizarbe B, Cerdan S, Brody L, et al The short-chain fatty acid acetate reduces appetite via a central homeostatic mechanism. Nat Commun. 2014;5:3611 Epub 2014/05/02. 10.1038/ncomms4611 24781306 PMC4015327

[33] Bergman EN. Energy contributions of volatile fatty acids from the gastrointestinal tract in various species. Physiol Rev. 1990;70(2):567–90. Epub 1990/04/01. 10.1152/physrev.1990.70.2.567 . 2181501

[34] den Besten G, van Eunen K, Groen AK, Venema K, Reijngoud DJ, Bakker BM. The role of short-chain fatty acids in the interplay between diet, gut microbiota, and host energy metabolism. J Lipid Res. 2013;54(9):2325–40. Epub 2013/07/04. 10.1194/jlr.R036012 23821742 PMC3735932

[35] McNeil NI. The contribution of the large intestine to energy supplies in man. Am J Clin Nutr. 1984;39(2):338–42. Epub 1984/02/01. 10.1093/ajcn/39.2.338 . 6320630

[36] Dinh DM, Ramadass B, Kattula D, Sarkar R, Braunstein P, Tai A, et al Longitudinal Analysis of the Intestinal Microbiota in Persistently Stunted Young Children in South India. PLoS One. 2016;11(5):e0155405 Epub 2016/05/27. 10.1371/journal.pone.0155405 27228122 PMC4881907

[37] Yatsunenko T, Rey FE, Manary MJ, Trehan I, Dominguez-Bello MG, Contreras M, et al Human gut microbiome viewed across age and geography. Nature. 2012;486(7402):222–7. Epub 2012/06/16. 10.1038/nature11053 22699611 PMC3376388

[38] Armougom F, Henry M, Vialettes B, Raccah D, Raoult D. Monitoring bacterial community of human gut microbiota reveals an increase in Lactobacillus in obese patients and Methanogens in anorexic patients. PLoS One. 2009;4(9):e7125 Epub 2009/09/24. 10.1371/journal.pone.0007125 19774074 PMC2742902

