# Supplementary material for: Gut microbiota profile of Indonesian stunted children and children with normal nutritional status
Source: PLoS One. 2021 Jan 26;16(1):e0245399. doi: 10.1371/journal.pone.0245399 (PMC7837488; doi:10.1371/journal.pone.0245399)
Supplement: S1 Table — (DOCX) [file pone.0245399.s002.docx]

S1 Table. Taxa that were different between sampling sites (Pandeglang [P] and Sumedang [Su]) and/or different between normal (N) or stunted (S) children when split up by sampling site.

| **taxa** | **difference between sites** | **difference between N and S** | **plot in Figure 4** |
| --- | --- | --- | --- |
|  | ***q*-value** | ***q*-value** |  |
| Erysipelotrichaceae UCG-004 | 3.6*10^-9^ | 4.6*10^-8^ | A |
| Sutterella | 4.3*10^-7^ | 4.3*10^-6^ | B |
| Anaeroplasma | 3.0*10^-5^ | 0.0037 | C |
| uncultured genus of Erysipelotrichaceae | 0.0011 | 7.2*10^-5^ |  |
| Ruminiclostridium 5 | 0.0011 | 0.020 | D |
| uncultured genus 1 of Clostridiales vadinBB60 group | 0.0013 | 0.002 |  |
| uncultured genus 2 of Clostridiales vadinBB60 group | 0.0016 | 0.005 | E |
| Campylobacter | 0.0020 | 0.084 |  |
| Ruminococcaceae UCG-008 | 0.0025 | 0.006 |  |
| uncultured genus of Mollicutes(NB1-n) | 0.0025 | 0.00014 |  |
| Lachnoclostridium | 0.0025 | 0.047 | F |
| Lachnospiraceae UCG-004 | 0.0025 | 0.046 | G |
| Ruminococcaceae UCG-003 | 0.0034 | 0.015 | H |
| Eubacterium hallii group | 0.0036 | 0.065 | I |
| uncultured genus of Rhodospirillaceae | 0.0045 | 0.086 |  |
| Brachyspira | 0.0047 | 0.019 |  |
| uncultured genus of Victivallales(vadinBE97) | 0.0059 | 0.0056 |  |
| Ureaplasma | 0.0070 | - |  |
| uncultured genus of Gastranaerophilales | 0.0087 | 0.099 |  |
| uncultured genus of Victivallaceae | 0.0091 | 0.086 |  |
| Eubacterium brachy group | 0.011 | - |  |
| Helicobacter | 0.018 | - |  |
| uncultured genus of Opitutae(vadinHA64) | 0.024 | - |  |
| Eubacterium fissicatena group | 0.024 | - |  |
| Victivallis | 0.026 | 0.092 |  |
| Clostridium innocuum group | 0.032 | - |  |
| uncultured genus of Prevotellaceae | 0.034 | - |  |
| Tyzzerella 4 | 0.034 | - |  |
| Allisonella | 0.036 | - |  |
| Eubacterium coprostanoligenes group | 0.037 | - |  |
| Bacteroides | 0.042 | - |  |
| Streptococcus | 0.047 | - |  |
| Catabacter | 0.052 | - |  |
| Ruminococcaceae UCG-004 | 0.056 | - |  |
| Christensenella | 0.060 | - |  |
| Actinobacillus | 0.069 | - |  |
| Lachnospiraceae UCG-008 | 0.073 | - |  |
| uncultured genus of Peptostreptococcaceae | 0.082 | - |  |
| Eisenbergiella | 0.086 | - |  |
| Ruminiclostridium 9 | 0.086 | - |  |
| Lachnospira | - | 0.037 |  |
| Senegalimassilia | - | 0.039 |  |
| Clostridium innocuum group | - | 0.049 |  |
| Alloprevotella | - | 0.057 |  |
| Ruminococcaceae.UCG.010 | - | 0.077 |  |
| Acetanaerobacterium | - | 0.083 |  |
| uncultured genus of Bacteroidales(S24-7 group) | - | 0.087 |  |
| uncultured genus of Ruminococcaceae | - | 0.087 |  |
| Lachnospiraceae UCG-003 | - | 0.089 |  |
| uncultured genus of Mollicutes(RF9) | - | 0.092 |  |
