# Supplementary material for: Gut microbiota profile of Indonesian stunted children and children with normal nutritional status
Source: PLoS One. 2021 Jan 26;16(1):e0245399. doi: 10.1371/journal.pone.0245399 (PMC7837488; doi:10.1371/journal.pone.0245399)
Supplement: S1 Fig — A. Unweighted UniFrac; B. Bray-Curtis, and C. Jaccard PCoA plots for the normal nutritional status (red) and stunted (blue) children. D: alpha-diversity indices Shannon index, observed OTUs, Faith’s phylogenetic diversity (PD), and evenness. All metrics q < 0.06. (DOCX) [file pone.0245399.s003.docx]

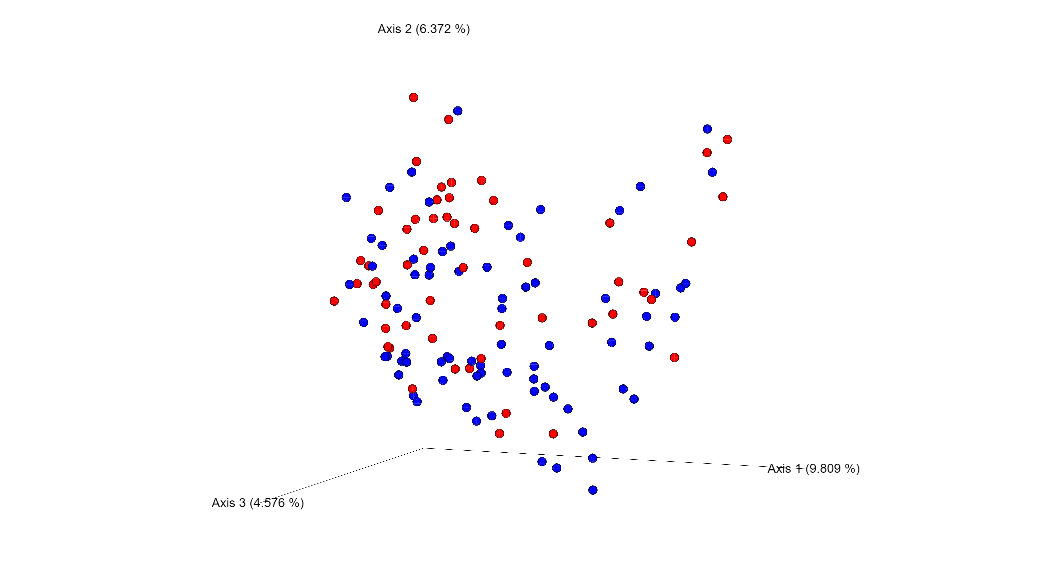

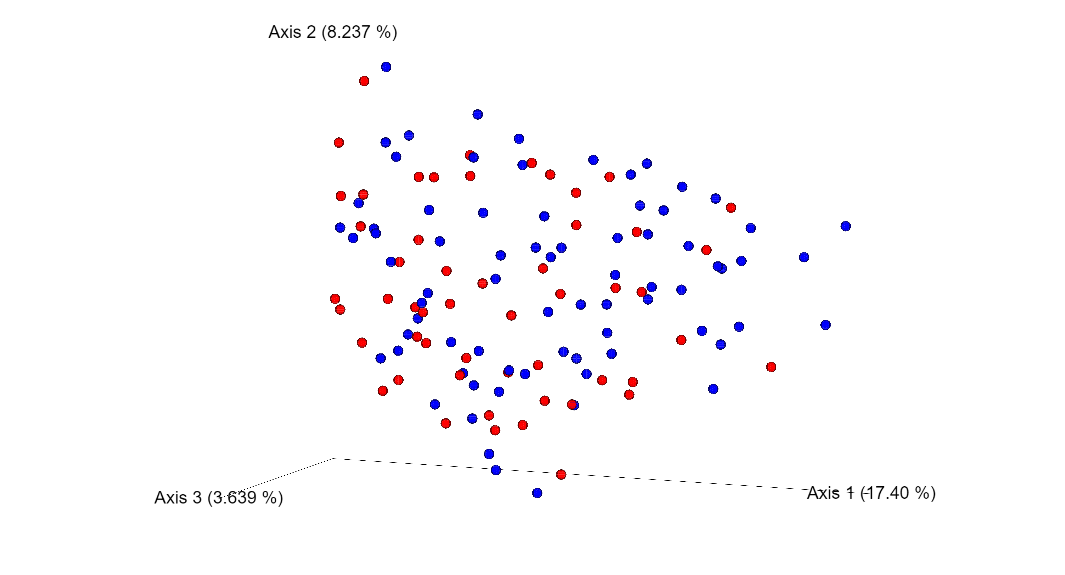
A B


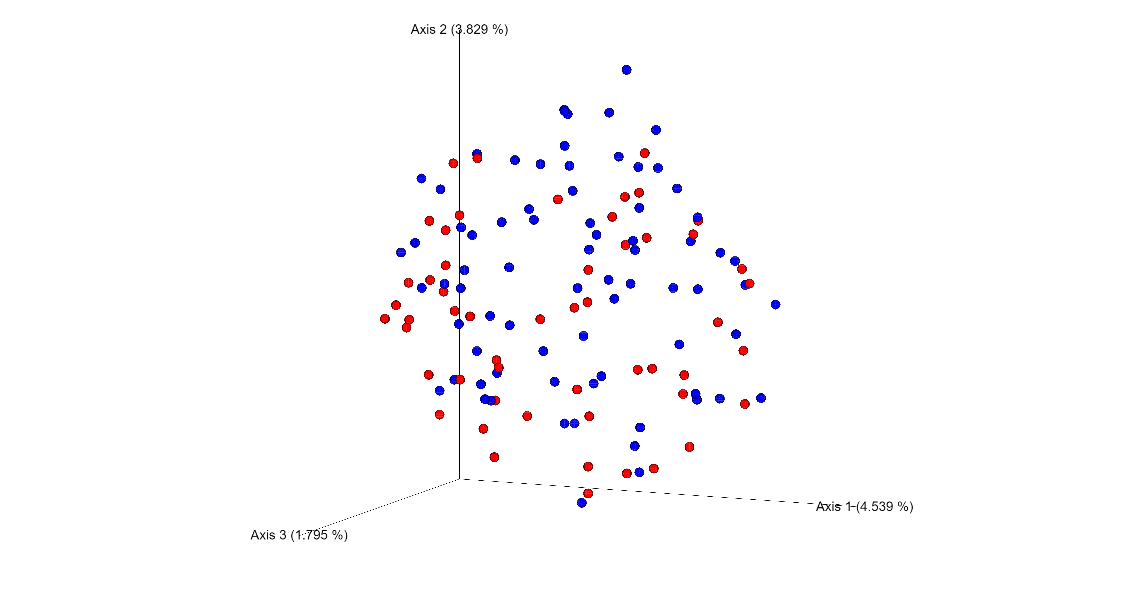


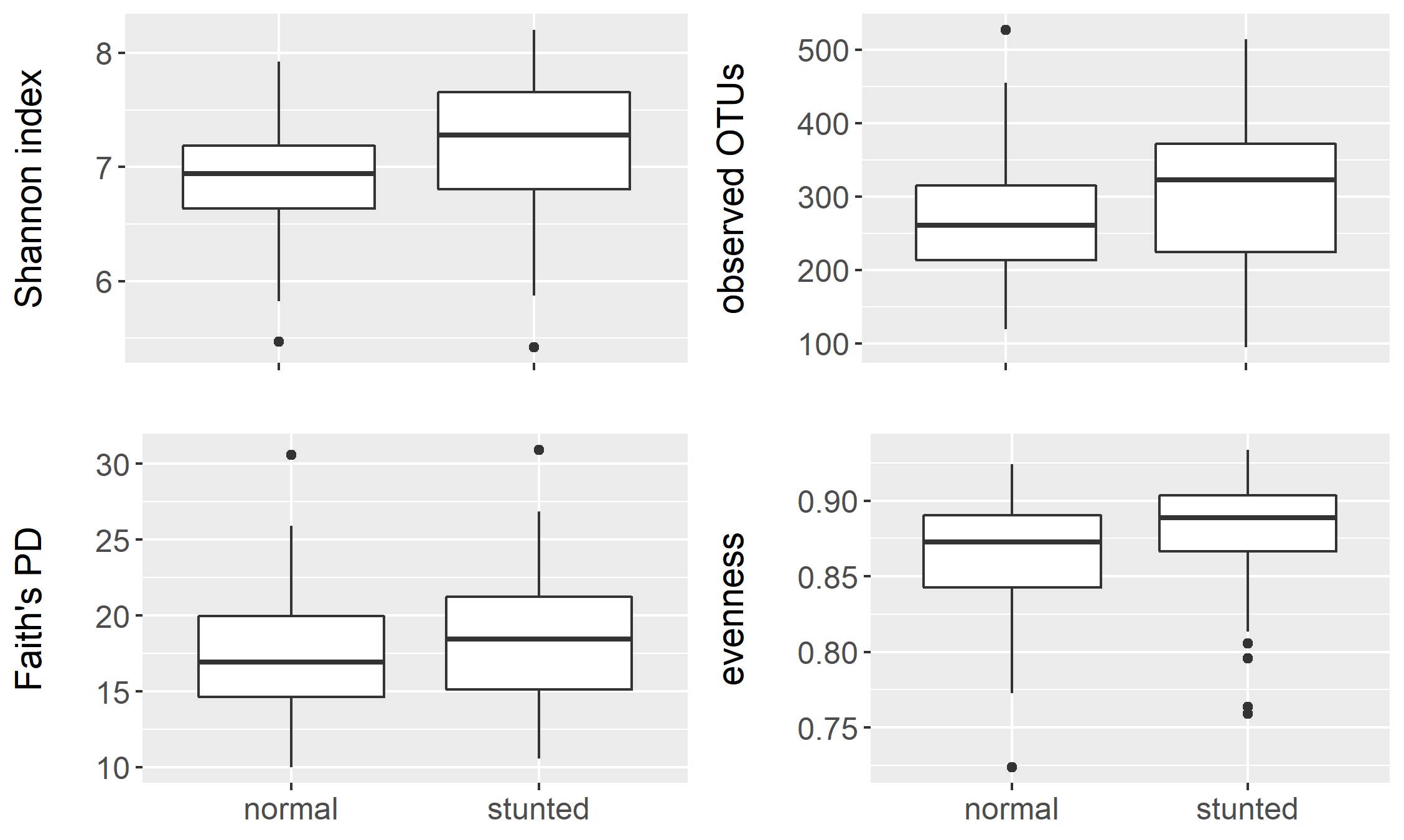
C D

S1 Fig. A. Unweighted UniFrac; B. Bray-Curtis, and C. Jaccard PCoA plots for the normal nutritional status (red) and stunted (blue) children. D: alpha-diversity indices Shannon index, observed OTUs, Faith’s phylogenetic diversity (PD), and evenness. All metrics *q* < 0.06.
