# Supplementary material for: Gut microbiota profile of Indonesian stunted children and children with normal nutritional status
Source: PLoS One. 2021 Jan 26;16(1):e0245399. doi: 10.1371/journal.pone.0245399 (PMC7837488; doi:10.1371/journal.pone.0245399)
Supplement: S2 Fig — A. Akkermansia, correlation coefficient -0.383; B. Pyramidobacter, correlation coefficient -0.271; C. Alistipes; correlation coefficient -0.265; D. Comamonas, correlation coefficient -0.233; E. Providencia, correlation coefficient -0.216. (DOCX) [file pone.0245399.s004.docx]

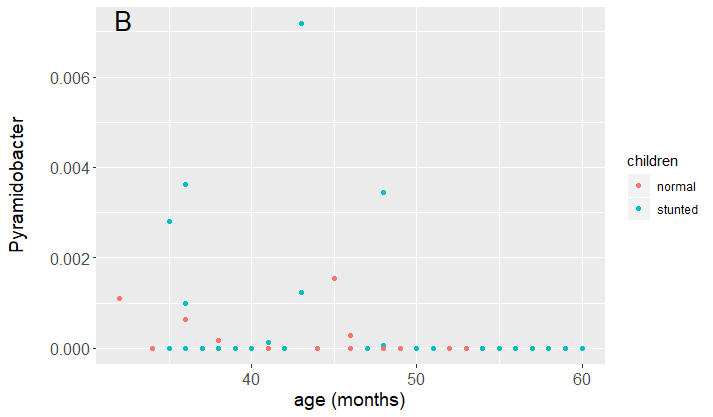

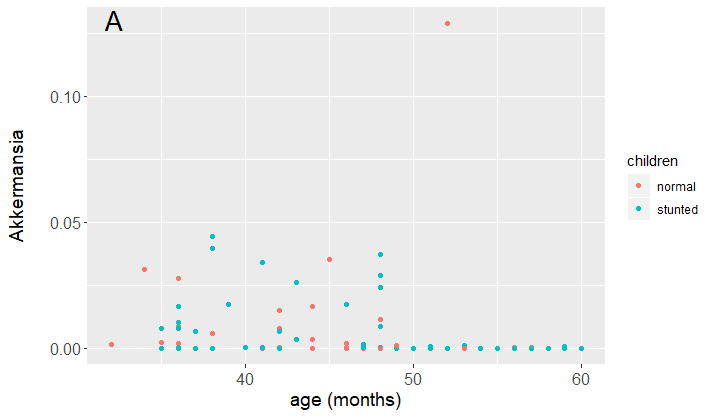


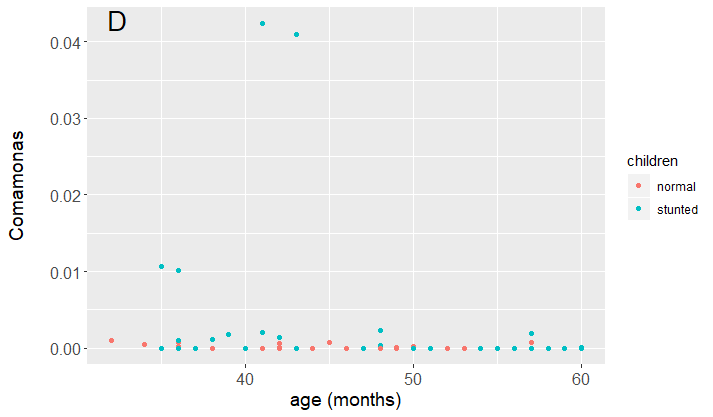

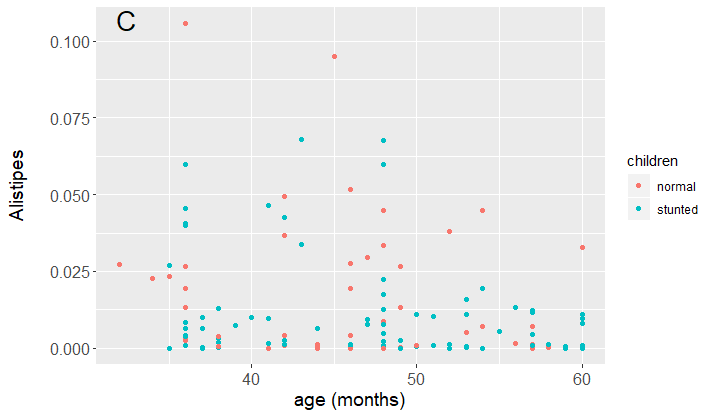


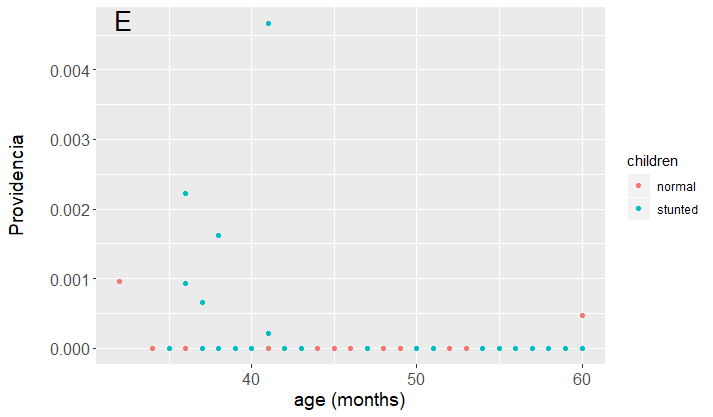


S2 Fig. Scatterplots of the five taxa that significantly negatively correlated with age. A. *Akkermansia*, correlation coefficient -0.383; B. *Pyramidobacter*, correlation coefficient -0.271; C. *Alistipes*; correlation coefficient -0.265; D. *Comamonas*, correlation coefficient -0.233; E. *Providencia*, correlation coefficient -0.216.
