# Supplementary material for: Gut microbiota profile of Indonesian stunted children and children with normal nutritional status
Source: PLoS One. 2021 Jan 26;16(1):e0245399. doi: 10.1371/journal.pone.0245399 (PMC7837488; doi:10.1371/journal.pone.0245399)
Supplement: S3 Fig — A. Ruminococcaceae UCG-014, correlation coefficient -0.282; B. uncultured genus of Mollicutes(RF9), correlation coefficient -0.280; C. Leuconostoc; correlation coefficient -0.267; D. Prevotella 9, correlation coefficient +0.238; E. uncultured genus of Gastranaerophilales, correlation coefficient -0.236; F. Desulfovibrio, correlation coefficient -0.232; G. Intestinimonas, correlation coefficient -0.220; H. Caproiciproducens, correlation coefficient -0.216; I. Prevotella 9, correlation coefficient +0.279; J. Leuconostoc, correlation coefficient +0.238; K. Alloprevotella, correlation coefficient +0.237; L. Bacteroides, correlation coefficient -0.237; M., correlation coefficient -0.21; N. Catenibacterium, correlation coefficient +0.220. (DOCX) [file pone.0245399.s005.docx]

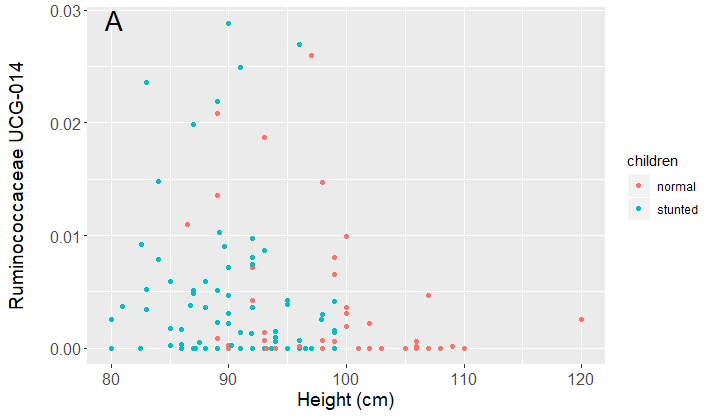

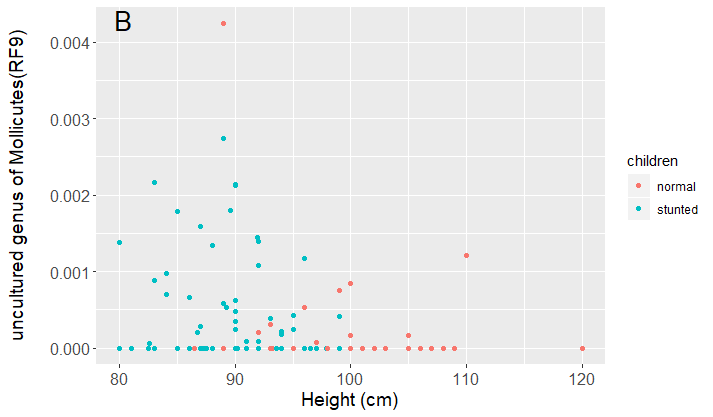

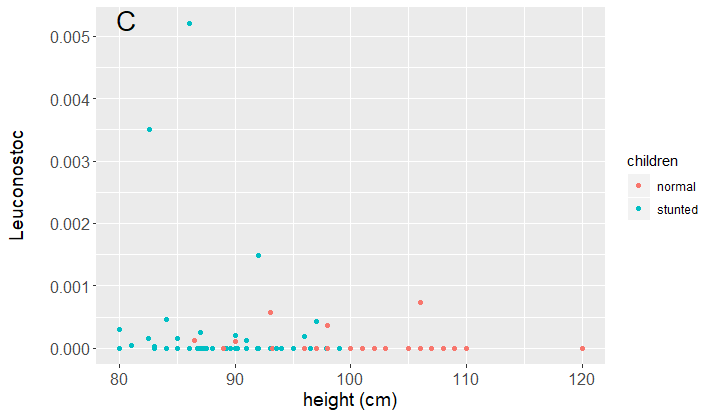

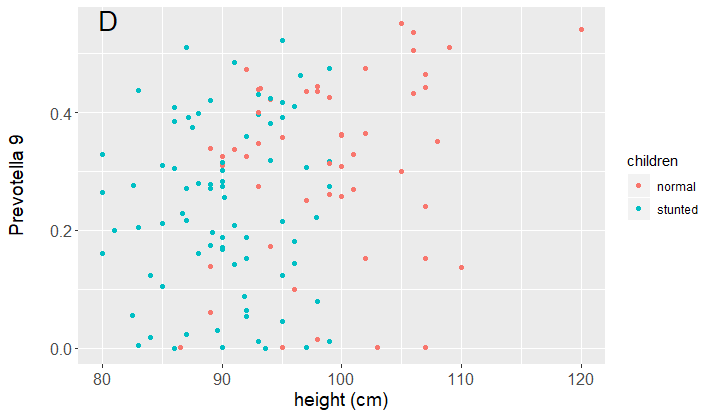

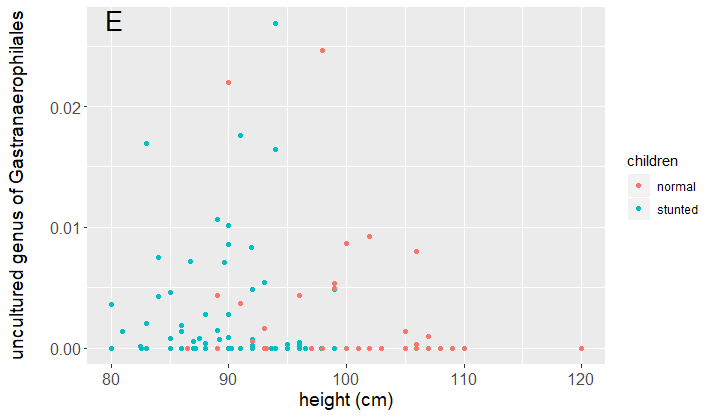

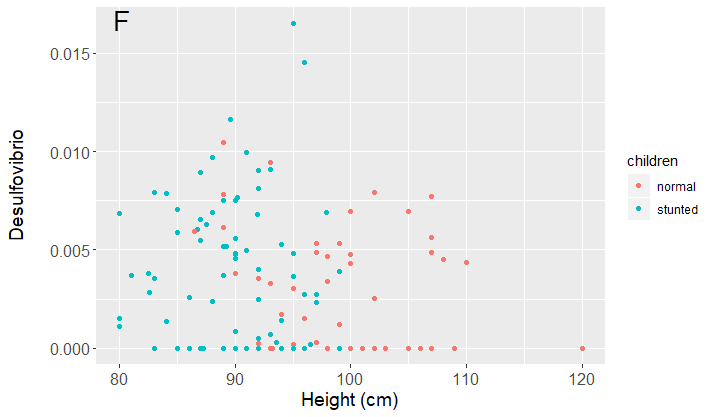


S3 Fig. Scatterplots of the taxa that are significantly correlated with height (A-H), weight (I) and BMI (J-M). A. *Ruminococcaceae* UCG-014, correlation coefficient -0.282; B. uncultured genus of Mollicutes(RF9), correlation coefficient -0.280; C. *Leuconostoc*; correlation coefficient -0.267; D. *Prevotella* 9, correlation coefficient +0.238; E. uncultured genus of Gastranaerophilales, correlation coefficient -0.236 ; F. *Desulfovibrio*, correlation coefficient -0.232; … *continued on next page* …


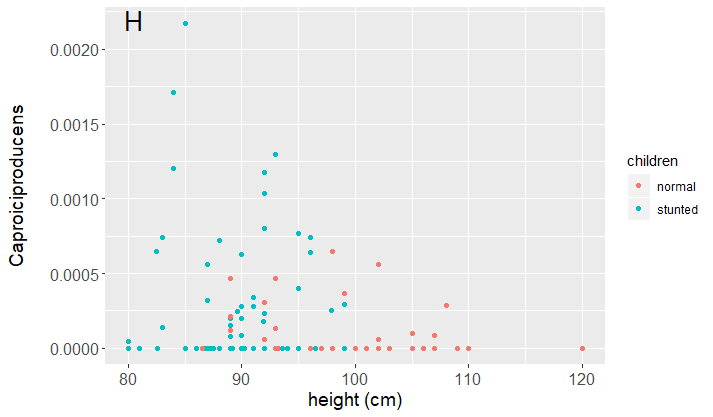

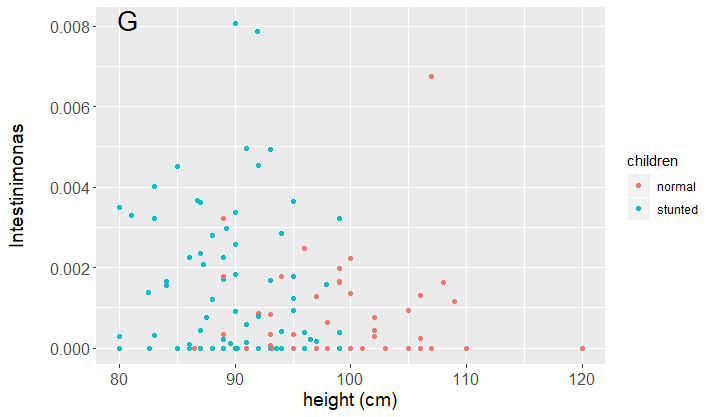


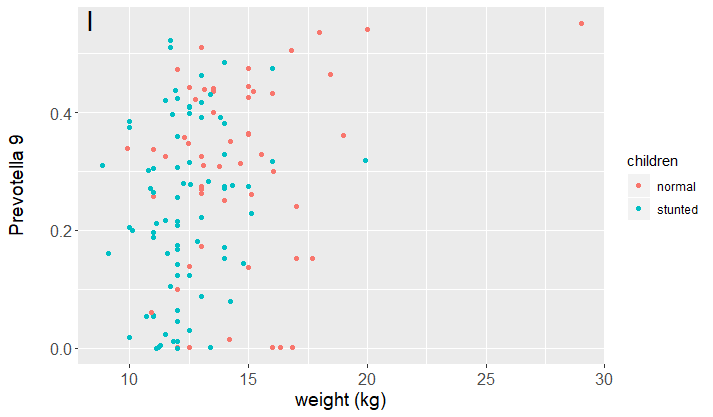

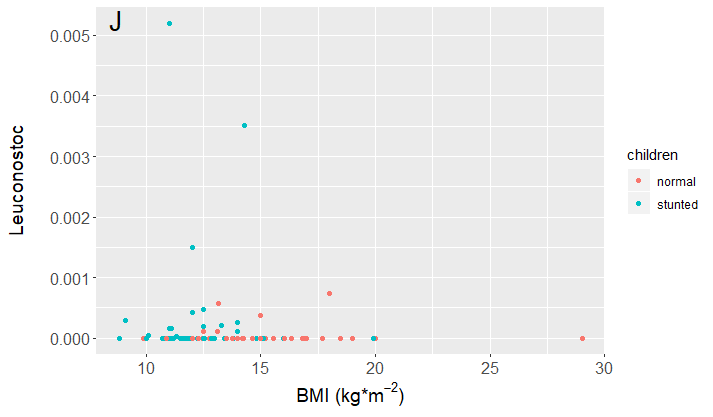

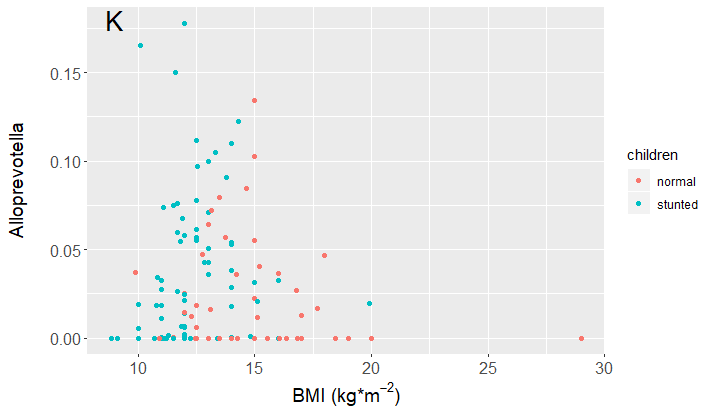

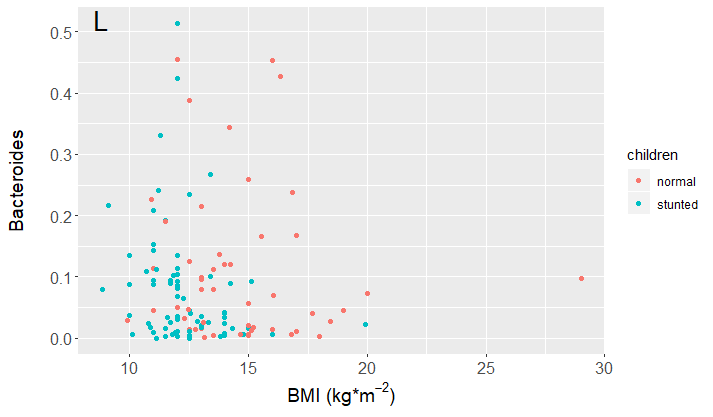

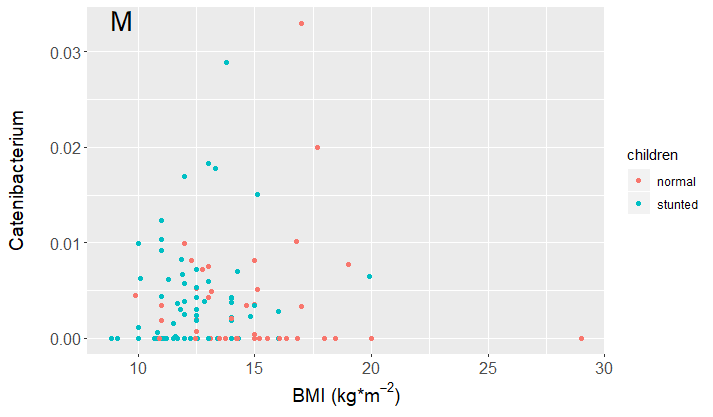


S3 Fig *continued*. Scatterplots of the taxa that are significantly correlated with height (A-H), weight (I) and BMI (J-M). …. G. *Intestinimonas*, correlation coefficient -0.220; H. *Caproiciproducens*, correlation coefficient -0.216; I. *Prevotella* 9 , correlation coefficient +0.279; J. *Leuconostoc*, correlation coefficient +0.238; K. *Alloprevotella*, correlation coefficient +0.237; L. *Bacteroides*, correlation coefficient -0.237; M. , correlation coefficient -0.21; N. *Catenibacterium*, correlation coefficient +0.220.
