# Supplementary figures and images for: Gut microbiota profile of Indonesian stunted children and children with normal nutritional status
Source: PLoS One. 2021 Jan 26;16(1):e0245399. doi: 10.1371/journal.pone.0245399 (PMC7837488; doi:10.1371/journal.pone.0245399)

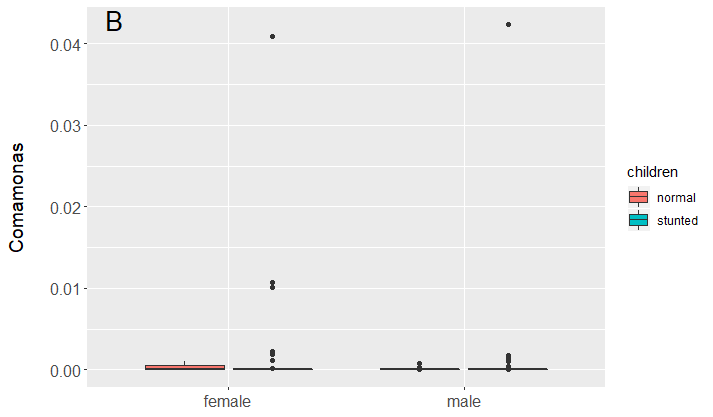


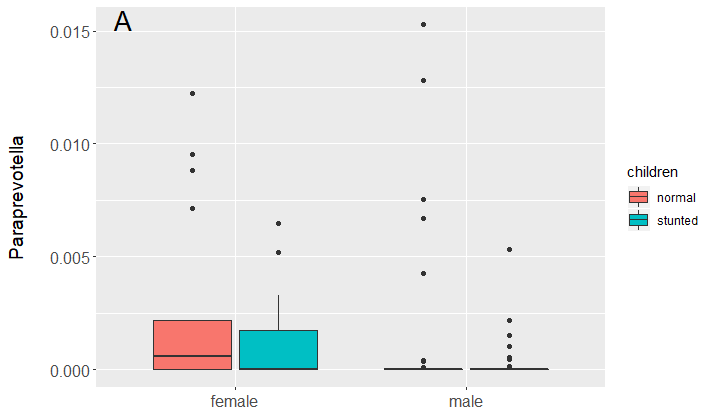


S4 Fig. Boxplots of the 2 taxa that are significantly correlated with gender.

Supplement: S4 Fig — (DOCX) [file pone.0245399.s006.docx]
