# Supplementary material for: Gut microbiota profile of Indonesian stunted children and children with normal nutritional status
Source: PLoS One. 2021 Jan 26;16(1):e0245399. doi: 10.1371/journal.pone.0245399 (PMC7837488; doi:10.1371/journal.pone.0245399)
Supplement: S1 Study — (PDF) [file pone.0245399.s007.pdf]

PROTOKOL STUDI

1. Jenis dan Desain Penelitian

Penelitian observasional ini menggunakan desain *Cross Sectional* (potong lintang) dengan pendekatan kuantitatif. Desain penelitian *cross-sectional* adalah penelitian yang dilakukan pada satu waktu dan satu kali untuk mencari hubungan antara variabel independen (faktor risiko) dengan variabel dependen (efek).

2. Waktu dan Lokasi Penelitian

Penelitian dilakukan selama 6 (enam) bulan tahun 2020 di desa lokus stunting di Kabupaten Pandeglang, Provinsi Banten yaitu Tegal Ongok dan Pasirkarang serta di Kabupaten Sumedang.

3. Populasi dan Sampel Penelitian

Populasi adalah semua anak usia balita umur 3-5 tahun di desa lokus stunting dan balita tidak stunting pada masing-masing kabupaten.

Kriteria Inklusi

a. Usia anak 3 – 5 tahun (36-60 bulan)
b. Tinggal di daerah pengamatan
c. Keadaan umum sehat ditetapkan oleh tenaga medis
d. Bersedia berpartisipasi dalam penelitian (*informent concern* ditandatangi ibunya)

Kriteria Eksklusi

a. Berusia di bawah < 36 bulan dan > 60 bulan
b. Tidak tinggal di daerah pengamatan
c. Tidak dalam keadaan sehat ditetapkan oleh tenaga medis
d. Tidak bersedia berpartisipasi dalam penelitian

Kriteria Drop Out

a. Tidak mengikuti atau berpartisipasi dalam pengukuran dan pengambilan sampel

4. Variabel Penelitian

Variabel yang diteliti adalah status gizi balita, karakteristik balita (usia, jenis kelamin, BBL, panjang lahir, riwayat imunisasi, riwayat diare, riwayat ISPA, asupan makanan), karakteristik ibu dan keluarga (usia ibu, pendidikan ibu, pekerjaan ibu, pekerjaan ayah, dan jumlah pendapat keluarga), lingkungan rumah (aspek komponen rumah, aspek sarana sanitasi, dan aspek perilaku penghuni), status mikrobiota usus.

Data diperoleh melalui pengukuran, wawancara menggunakan kuesioner dan observasi langsung menggunakan cek list.

Definisi masing-masing variabel yang akan diteliti adalah:

4.1 Status gizi balita

Adalah pengukuran antropometri stunting diukur berdasarkan parameter panjang/tinggi badan menurut usia dibandingkan dengan standar antropometri WHO 2005 dan Kepmenkes RI No. 1995/MENKES/SK/XII/2010.

Status nutrisi dari setiap anak yang terlibat dalam penelitian ini ditetapkan berdasarkan rekomendasi WHO (status gizi Z-score), panjang dibagi umur height (Z-score 1); berat dibagi umur (Z-score 2) dan berat dibagi tinggi (Z-score 3).

Sebuah kuesioner terstruktur digunakan dalam wawancara langsung berdasarkan informasi ibu untuk mendapatkan data informasi sosiodemografi dan pola makan. Selain itu usia dan pengukuran antropometri (tinggi, berat) didasarkan pada ketentuan regulasi Kementrian Kesehatan Republik Indonesia. Untuk stunting, batasan tinggi berdasarkan usia adalah : '*severely stunted*' (<-3 SD); '*stunted*' (-3 SD to < -2 SD); '*normal*' (-2 SD to +3 SD); '*tall*' (> +3 SD). Lebih jauh lagi, untuk mendapatkan gambaran status nutrisi anak, diklasifikan berdasarkan kategori *weight-for-height*: '*severely wasted*' (<-3 SD); '*wasted*' (-3 SD to < -2 SD); '*normal*' (-2 SD to +1 SD); '*possible risk of overweight'* (+1 SD to +2 SD); '*over weight'* (> +2 SD to +3 SD); '*obese*' (> +3 SD).

4.2 Karakteristik Balita

a. Usia balita adalah usia atau lama waktu hidup responden balita dihitung dalam bulan sejak lahir sampai ulang bulan terakhir.

b. Jenis Kelamin adalah identitas yang dibedakan secara fisik berdasarkan organ genitalis eksternal.

c. Berat badan lahir (BBL) adalah bobot badan bayi pada saat dilahirkan dalam gram yang tercatat dalam KMS.

d. Panjang lahir adalah panjang badan bayi pada saat dilahirkan dalam sentimeter yang tercatat dalam KMS.

e. Riwayat imunisasi adalah lengkap tidaknya anak mendapatkan imunisasi yang dijadwalkan sesuai dengan usianya.

f. Riwayat Diare adalah adanya riwayat terkena diare dalam dua minggu terakhir.

g. Riwayat ISPA adalah adanya riwayat penyakit infeksi saluran pernafasan atas (batuk, pilek, dan demam) dalam dua minggu terakhir.

h. Asupan makanan adalah jumlah dan jenis makanan balita dalam waktu 7 hari terakhir,diukur dengan food record dan dihitung dengan nutrisurvey.

4.3 Data Anthropometri

4.3.1 Identitas Ibu

Nama :

Alamat :

Kecamatan :

Desa :

a. Tinggi badan Ibu : ……………….. cm

b. Berat Badan Ibu : ………………… kg

c. IMT : Kurus (<17-18,4)/Normal(18,5-25)/Gemuk (25,1->27)

c. Usia ibu : …………………tahun

d. Pendidikan ibu : tidak sekolah / SD / SMP / SMA / Sarjana

e. Pekerjaan ibu : tidak bekerja / bekerja, sebutkan…………….(Formal/Informal)

h. Pendidikan ayah : tidak sekolah / SD / SMP / SMA / Sarjana

i. Pekerjaan ayah : tidak bekerja / bekerja, sebutkan………………….(Formal/Informal)

j. Jumlah pendapat keluarga : < UMR / > UMR (sebutkan jumlahnya Rp……………….)

k. Status Pernikahan : Menikah/janda/cerai

l. Usia pertama menikah ibu : ……………….th

4.3.2 Identitas Baduta

a. Nama : …………..

b. Usia baduta : …………. bulan

c. Jenis Kelamin : laki-laki/ perempuan

d. Persalinan ditolong oleh : nakes/bukan nakes

d. Berat badan lahir (BBL) : ………gram

e. Panjang lahir *:* ………….cm

f. Riwayat imunisasi : lengkap / tidak lengkap

g. Riwayat Diare adalah (dalam dua minggu terakhir) : ada / Tidak ada

h. Riwayat ISPA (dalam dua minggu terakhir) : ada / Tidak ada

1).Status gizi baduta

BB : ……..kg

PB : ………cm

Status gizi : …........................

Status stunting : …........................

4.4 Profil mikrobiota usus

a. Cara pengambilan sampel feses

Feses diambil dari balita stunting, total 200 dari 2 lokasi, Pandeglang dan Sumedang. Feses ditampung dengan menggunakan wadah plastik, dan tidak mengenai air seni dan jamban. Feses segera dipindahkan ke dalam kontainer feses menggunakan sendok feses, dan disimpan dalam kotak suhu dingin menggunakan *ice pack*. Feses segera ditransportasikan ke laboratorium. Feses diberikan buffer untuk mempertahankan DNA supaya tidak rusak.

b. Cara pemeriksaan

Mikrobiota pada feses dianalisis profilnya dengan menggunakan *Next Generation Sequencing* (NGS) di Masstricht University. Feses juga dianalisis mikrobiotanya secara Total plate count, isolasi dan identifikasi sesuai metode standar mikrobiologi, sedangkan identifikasi telur cacing pada feses dilakukan dengan metode Kato Kantz di laboratorium Mikrobiologi Parasitologi Universitas YARSI dan UKI.

5. Metode Analisis Data

a. Uji keragaman mikrobiota Quantitative Insights Into Microbial Ecology (QIIME) digunakan untuk pengelompokan hasil bacaan (*reads*) ke Unit Operasional taksonomi (OTU) pada 99% urutan kesamaan terhadap database *Greengenes*, Q30 = 75,9%, kepadatan kluster 808K / mm^2. α-Diversity (keragaman dalam sampel) dianalisis menggunakan metrik keragaman: Keragaman filogenetik (PD-Whole-Tree), indeks Shannon, Chao dan UTO yang Teramati. Pada β-diversity, UniFrac (weigthed dan unweighted) divisualisasikan menggunakan 3D-graph *principal coordinate analysis* (PCoA), menggunakan alat PhyloToAST. β-diversity UniFrac dapat dibagi menjadi *weight* yang secara langsung memperhitungkan perbedaan dalam kelimpahan relatif yang dapat menghasilkan hasil yang berbeda namun saling melengkapi dan *unweight* (ada atau tidak adanya OTU) adalah jarak antara dua komunitas.

b. Selain analisis menggunakan paket sistem QIIME, analisis statistik juga dilakukan dengan menggunakan perangkat lunak SPSS 22. (IBM SPSS Statistics for windows versi 22.0 Armonk, NY; IBM Corp). Uji Shapiro-Wilk dilakukan untuk menguji normalitas distribusi semua variabel kuantitatif yang diteliti. Apabila variabel tidak mengikuti distribusi normal, metode non-parametrik dipilih untuk analisis data lebih lanjut. Metode nonparametrik Mann Whitney, Kruskall wallis dan Spearman dalam R (function cor.test) pada korelasi antara beberapa parameter dalam profil mikrobiota.

UNIVERSITAS
YARSI
LEMBAGA PENELITIAN
RESEARCH INSTITUTE

KOMITE ETIK PENELITIAN
LEMBAGA PENELITIAN UNIVERSITAS YARSI

KETERANGAN LOLOS *KELAYAKAN ETIK PENELITIAN*

No: 004/KEP-UY/BIA/I/2020

Setelah mengkaji secara mendalam dan komprehensif proposal berjudul: **Profil mikrobiota dan metabolit Short Chain Fatty Acid (SCFA), status imun, Lipobinding protein (LBP), serta *hygiene practice* pada balita sehat dan stunting.**

Nama peneliti utama : ***Dian Widiyanti, S.Si., M.Si., Ph.D***

Nama institusi : ***Fakultas Kedokteran Universitas YARSI***

Anggota : ***1. Prof. Ir. Ingrid Suryanti Surono, M.Sc., Ph.D***
(Program Studi Teknologi Pangan, Fakultas Teknik, Universitas Bina Nusantara)
2. Dr. med. dr. Abraham Simatupang, M.Kes
(Fakultas Kedokteran, Universitas Kristen Indonesia)

dengan penuh tanggung jawab menyatakan bahwa usulan penelitian telah memenuhi persyaratan Etik dan disetujui untuk dilaksanakan.

Jakarta, 09 Januari 2020

Ketua,

UNIVERSITAS YARSI
LEMBAGA PENELITIAN
RESEARCH INSTITUTE

Prof. dr. Hj. Qomariah RS, MS., PKK., AIFM

NIK: 531111179022
