# Supplementary material for: Gut microbiota profile of Indonesian stunted children and children with normal nutritional status
Source: PLoS One. 2021 Jan 26;16(1):e0245399. doi: 10.1371/journal.pone.0245399 (PMC7837488; doi:10.1371/journal.pone.0245399)
Supplement: S2 Study — (PDF) [file pone.0245399.s008.pdf]

RESEARCH PROTOCOL

1. Type and Research Design

A cross sectional study design with a quantitative approach will be conducted. Cross-sectional research design is a one-time research to find a relationship between the independent variable (risk factor) and the dependent variable (effect).

2. Time and Location of Research

Research will be conducted for six months in 2020 at Banten Regency (namely Tegal Ongok and Pasirkarang) and Sumedang Regency.

3. Population and Research Sample

The population is childrens between three and five years old in the village of stunting locus. The sampling method to be used in this research was quota sampling, namely 50 stunting and 50 healthy childrens from each Regency.

Inclusion criteria

a. Children age : 3 – 5 years old (36-60 months)
b. Live in research area
c. Apparently healthy children, after examination by physician
d. Involved in research study (informed concent signed by parent or guardian)

Exclusion criteria

a. Children age < 36 month and > 60 month
b. Live outside research area
c. Unhealthy, after examination by physician

Drop Out Criteria

a. Do not follow or participate in antrophometric measurement or collecting fecal sample.

4. Research Variables

The variables studied will be the nutritional status of children between three and five years old, the characteristics of these children (age, sex, LBW, birth length, history of immunization, history of diarrhea, history of upper respiratory tract infection, food intake), mother and family characteristics (mother's age, mother's education, mother's occupation, father's occupation), home environment (aspects of the components of the house, aspects of sanitation facilities, aspects of occupant behavior), composition of the intestinal microbiota.

Data will be obtained through measurements, interviews using a questionnaire and direct observation using a check list.

The definition of each variable :

4.1 The nutritional status of children under five

Stunting anthropometric measurements are measured based on parameters of length / height according to age compared to the WHO anthropometric standards 2005 and the Indonesian Minister of Health Decree No. 1995 / MENKES / SK / XII / 2010.

The nutritional status of each children included in this study will be quantified using the WHO recommended three nutritional Z-scores namely, height for age (referred to in this study as Z-score 1); weight for age (referred as Z-score 2) and weight for height (referred as Z-score 3).

A structured questionnaire was used for face-to-face interviews with the respective child’s mother to collect sociodemographic information. In addition, age and anthropometric measurements (height, weight) based on Department of Health Ministry of Indonesia Regulation will be recorded. For stunting, the thresholds for height-for-age are: ‘severely stunted’ (<-3 SD); ‘stunted’ (-3 SD to < -2 SD); ‘normal’ (-2 SD to +3 SD); ‘tall’ (> +3 SD). Furthermore, in order to obtain an overall measure of the nutritional status of these children, the children will be classified in weight-for-height categories: ‘severely wasted’ (<-3 SD); ‘wasted’ (-3 SD to < -2 SD); ‘normal’ (-2 SD to +1 SD); ‘possible risk of overweight’ (+1 SD to +2 SD); ‘over weight’ (> +2 SD to +3 SD); ‘obese’ (> +3 SD)

4.2 Characteristics of Children

a. “Balita age” is the children age from births until the last birthday.

b. Gender is a physically differentiated identity based on external genital organs.

c. Birth weight (LBW) is the weight of the baby at birth in grams recorded in their health record

d. Birth length is the length of the baby's body at birth in centimeters as recorded in their health record.

e. Immunization history is the immunization scheduled, due to the children age.

f. History of diarrhea is a history of diarrhea in the last two weeks.

g. History of upper respiratory infection (cough, cold, and fever) in the last two weeks.

h. Food intake is the amount and type of food under five years of age within the last 7 days, measured by food records and calculated by nutritional surveys.

4.3 Anthropometric data

4.3.1 Mother identity

Name :

Addres :

District :

Village :

a. Mother height : ……………….. cm

b. Mother weight : ………………… kg

c. BMI : thin (<17-18,4)/Normal(18,5-25)/obese (25,1->27)

c. Mother age : …………………year

d. Mother education : none / elementary / yunior / senior / bachelor

e. Mother job : none / work,…………….(Formal/Informal)

h. Father education : none /elementary / yunior / senior / bachelor

i. Father job : none / work, ………………….(Formal/Informal)

j. Income : < RMW / > RMW (nominal…..)

k. Marriage status : marriage / widow / divorce

l. Mother age when marriage : ………………year

4.3.2 Children Identity

a. Name : …………..

b. Children age : ………….month

c. Gender : boy/grils

d. Partus help by : medician/non medician

d. Birth weight : ………gram

e. Birth height : …………cm

f. Immunization history : complete / not complete

g. Diarrhea history (within 2 weeks) : yes / no

h. upper respiratory tract infection (within 2 weeks) : yes / no

1) Nutritional status

Body Weight : ……..kg

Height: ………cm

Nutritional status : ....

Stunting status : …....................

4.4 Intestinal microbiota profile

a. Stool sampling method

Stool samples will be taken from all childrens that are screened, with a total of 200 from 2 locations, Pandeglang and Sumedang. Stools will be collected using pot stool containers, and

not include urine and latrines. The stools are immediately transferred to a stool container using a spoon, and stored in a cold temperature box using an ice pack. Stools will then immediately be transported to the laboratory. Stool samples will be buffered to keep DNA from being damaged.

b. Analysis

The microbiota profile in stool samples will be analyzed using Next Generation Sequencing (NGS) at Maastricht University, by sequencing the V3-V4 region of the 16S rRNA gene. Stools samples will also be analyzed for microbiota composition using total plate count, isolation and identification according to standard microbiological methods, while the identification of worm eggs in feces will be carried out by the Kato Kantz method in the YARSI and UKI University Parasitology Microbiology Laboratory.

5. Data Analysis Methods

a. The quantitative Insights Into Microbial Ecology (QIIME) pipeline will be used to study microbiota diversity based on grouping of the Taxonomic Operational Unit (OTU) reads at 99% similarity with comparison against the Greengenes database, Q30 = 75.9%, cluster density 808K / mm2. α-Diversity (diversity within the sample) will be analyzed using the diversity metric: Phylogenetic diversity (PD-Whole-Tree), Shannon index, Chao and Observed UTO. For β-diversity, UniFrac (weigthed and unweighted) will visualized using 3D-graph principal coordinate analysis (PCoA), using the Emperor tool. β-diversity of UniFrac can be divided into weighted (which directly account for differences in relative abundance) and unweighted (presence or absence of OTU) is the distance between two communities.

b. Apart from analysis using the QIIME system, statistical analysis will also be performed using SPSS (IBM SPSS Statistics for windows version 22.0 Armonk, NY; IBM Corp). The Shapiro-Wilk test will be conducted to test the normality of the distribution of all quantitative variables studied. If the variables do not follow a normal distribution, a non-parametric method is chosen for further data analysis. Nonparametric methods Mann Whitney, Kruskall wallis and Spearman in R (function cor.test) will be used to study the correlation between several parameters in the microbiota profile.

UNIVERSITAS YARSI
LEMBAGA PENELITIAN
RESEARCH INSTITUTE

RESEARCH ETHICS COMMITTEE
RESEARCH INSTITUTE YARSI UNIVERSITY

ETHICAL CLEARANCE

No: 004/KEP-UY/BIA/I/2020

The Research Ethics Committee, Universitas YARSI has learned and decided that the research proposal entitled:

Microbiome profile and Short Chain Fatty Acid (SCFA) metabolite, immunity status, Lipobinding protein, also hygiene practice of healthy and stunted toodlers.

Lead Researcher	: ***Dian Widiyanti, S.Si., M.Si., Ph.D***
Affiliation	: ***Faculty of Medicine, UniversitasYARSI, Jakarta***
Researcher Member	: ***1. Prof. Ir. Ingrid Suryanti Surono, M.Sc., Ph.D (Food Technology Study Program, Faculty of Engineering, Universitas Bina Nusantara, Jakarta).*** ***2. Dr. med. dr. Abraham Simatupang, M.Kes (Faculty of Medicine, Universitas Kristen Indonesia)***

using human specimen is approved as long as the research is conducted properly on the basis of respect for human dignity and human rights and refers to scientific and moral ethics.

Jakarta, January 09, 2020
Chairman,

UNIVERSITAS YARSI
LEMBAGA PENELITIAN
RESEARCH INSTITUTE

Prof. dr. Hj. Qomariah RS., MS., PKK., AIFM
NIP: 531111179022
